# *Staphylococcus epidermidis* DnaK alters biofilm formation and proteome in *Staphylococcus aureus* CIP 107093

**DOI:** 10.3389/fmicb.2025.1705130

**Published:** 2026-02-18

**Authors:** Clara Kayser, Karen Druart, Eleonore Bouscasse, Mariette Matondo, Andréa Chane, François Hoh, Anne Groboillot, Corinne Barbey, Annabelle Merieau, Xavier Latour, Pierre Soule, Estelle Mühle, Vic Norris, Yoan Konto-Ghiorghi

**Affiliations:** 1 Research Unit Bacterial Communication and Anti-infectious Strategies (CBSA, UR4312), University of Rouen Normandie, Evreux, France; 2 Paris Cité, CNRS USR2000, Unité de Spectrométrie de Masse pour la Biologie, Plateforme de protéomique, Paris, France; 3 Centre de Biochimie Structurale, CNRS UMR 5048-INSERM 1054, University of Montpellier, Montpellier, France; 4 NanoTemper Technologies GmbH, Munich, Germany; 5 Institut Pasteur, Université Paris Cité, Collection of Institut Pasteur (CIP), Paris, France

**Keywords:** DnaK, biofilm, *Staphylococcus epidermidis*, *Staphylococcus aureus*, skin microbiota, proteostasis

## Abstract

*Staphylococcus aureus* and *Staphylococcus epidermidis*, two Gram-positive bacteria of the human skin microbiota, form biofilms that contribute to dysbiosis and inflammatory skin diseases such as psoriasis and atopic dermatitis. The human calcitonin gene-related peptide (CGRP), involved in skin inflammation, was previously shown to enhance the virulence of *S. epidermidis* MFP04. We previously observed a significant increase in the level of the molecular chaperone DnaK/Hsp70 in the secretome of CGRP-activated *S. epidermidis*. Here, we investigated the role of recombinant *S. epidermidis* DnaK in biofilm formation in both *S. aureus* and *S. epidermidis*. DnaK modulates biofilm formation in a strain-dependent manner. In commensal strains (*S. aureus* MFP03 and *S. epidermidis* MFP04), it is associated with an increase in biofilm biomass. In contrast, it significantly reduces biofilm formation in the clinical *S. aureus* strain CIP 107093. Point mutations in the substrate-binding domain (SBD) and nucleotide-binding domain (NBD) of DnaK differentially affect its modulation of biofilm formation. Specifically, only the mutation in the SBD abolishes the biofilm reduction observed in CIP 107093, while the NBD mutation results in a milder effect. Notably, these mutations have no significant impact on DnaK-induced biofilm changes in strains where DnaK promotes biofilm formation. Proteomic analyses of *S. aureus* CIP 107093 reveal that DnaK alters the *S. aureus* biofilm proteome, stabilizing protein degradation components and downregulating key biofilm regulators. These findings highlight the cross-species regulatory potential of *S. epidermidis* extracellular DnaK in the skin microbiota.

## Introduction

The skin microbiota includes a large diversity of bacteria (Harris-Tryon and Grice, 2022; Hrestak et al., 2022), including the common commensal Gram-positive bacterial species *Staphylococcus aureus* and *Staphylococcus epidermidis* (Bier and Schittek, 2021). *S. aureus* is often associated with skin disorders, such as psoriasis and atopic dermatitis (AD) (Bier and Schittek, 2021). During AD inflammation, a dysbiosis of the skin microbiota occurs that leads, in particular, to increased *S. aureus* colonization at the expense of *S. epidermidis* (Brown and Horswill, 2020). Supporting the correlation between *S. aureus* and disease severity, the abundance of *S. aureus* in patients with AD was found to be 70% on lesional skin versus only 39% on healthy skin of the same patient (Hrestak et al., 2022). *S. epidermidis* is part of the healthy skin ecosystem, where it prevents microbiota disequilibrium and contributes to skin homeostasis (Severn and Horswill, 2022). *S. epidermidis* prevents *S. aureus* biofilm formation by secreting bacteriocins, metalloproteinases, and quorum sensing (QS) molecules that regulate the production of the antimicrobial peptide epidermin (Kies et al., 2003). However, emerging evidence shows that colonization by certain specific strains of *S. epidermidis* can also damage the skin (Brown and Horswill, 2020). Hence, the ability of *S. epidermidis* and *S. aureus* to form biofilms can be associated with chronic infections (Otto, 2009) and skin dysbiosis, as in AD (Hrestak et al., 2022), where such biofilms intensify lesions and inflammation (Brown and Horswill, 2020).

Biofilms are complex structures that protect bacterial cells from environmental changes, nutrient-deficient conditions, the immune system, and antibiotics (Otto, 2009). Microbial biofilms contribute to the emergence of multi-drug resistant (MDR) microorganisms, limiting antibiotic accessibility (Assefa and Amare, 2022) and facilitating the horizontal transfer of resistance genes (Maree et al., 2022). Exposure of biofilms to the host immune system also triggers chronic inflammation (Otto, 2009; Lee et al., 2022). The biofilm matrix is composed of extracellular DNA (eDNA), polysaccharides, and proteins (Porter et al., 2022). Extracellular DNA is released by the autolysis of bacterial cells into the biofilm matrix (Foster et al., 2014), where it plays a key structural role (Secchi et al., 2022). In staphylococcal species, polysaccharides, primarily the poly-N-acetyl glucosamine (PNAG) polysaccharide, also known as polysaccharide intercellular adhesin (PIA), help determine the surface properties and persistence of the biofilm (Nguyen et al., 2020). In *S. aureus*, matrix proteins include accumulation-associated proteins (Aap), fibrinogen-binding protein (FnBP) A and B, extracellular matrix-binding protein (Embp), amyloid fibers, surface-binding proteins, including SasG and staphylococcal protein A (Spa), clumping factor B (ClfB), extracellular adherence protein (Eap), and *α*-toxin (α-hemolysin), which targets the host (Foster et al., 2014; Yonemoto et al., 2019; Caiazza and O'Toole, 2003). These *S. aureus* proteins are involved in biofilm formation, surface or host adhesion, and interactions with other matrix components (Foster et al., 2014). Finally, *S. aureus* produces a family of proteins called phenol-soluble modulins (PSMs) that are involved in biofilm formation and immune cell death (Peschel and Otto, 2013). These PSMs are amyloid proteins and are essential for biofilm formation and its stabilization (Peschel and Otto, 2013; Schwartz et al., 2012). *S. aureus* also expresses numerous proteases involved in biofilm structuring and dispersion (Schilcher and Horswill, 2020). Some of these proteases have the ability to degrade the cutaneous barrier, such as the exfoliative toxin A and B (ETA and ETB), which are epidermolytic toxins. ETA is a serine protease that hydrolyzes desmoglein-1 (Dsg1) within desmosomes (Hanakawa and Stanley, 2004). Located in the deeper layers of the epidermis, Dsg1 degradation leads to the development of staphylococcal scalded skin syndrome (SSSS) (Ladhani, 2001).

The skin is a major neuroendocrine organ of the human body (Slominski et al., 2022). Neurotransmitters and neuropeptides are released in the skin to regulate stress responses. Microorganisms that colonize the skin can interact with these neuropeptides (Hrestak et al., 2022; N'Diaye et al., 2016). The calcitonin gene-related peptide (CGRP) is a peptide expressed in sensory skin fibers (Kim and Granstein, 2021) and is involved in nociception and vasodilation. CGRP also plays a role in regulating T cells, B cells, and dendritic cells, as well as in mediating skin inflammation (Kim and Granstein, 2021). We previously reported that CGRP increases the adherence and cytotoxicity of *S. epidermidis* MFP04 on HaCat keratinocytes (N'Diaye et al., 2016). A chaperone, DnaK, was detected in the secretome of CGRP-activated *S. epidermidis* and was identified as a CGRP-binding protein; however, the role of DnaK in adherence and cytotoxicity concerning HaCat cells was unknown.

DnaK is a molecular chaperone of the heat shock protein family involved in the folding of newly synthesized proteins and the refolding of misfolded proteins (Schneider and Antes, 2023). The DnaK amino acid sequences of *S. epidermidis* MFP04 and *S. aureus* MFP03 are 96% identical. DnaK contains an N-terminal nucleotide-binding domain (NBD) and a C-terminal substrate-binding domain, which is divided into two subdomains (SBD-*α* and SBD-*β*) (Schneider and Antes, 2023). DnaK recognizes exposed hydrophobic residues in denatured proteins (Gragerov et al., 1994; Sekhar et al., 2015). The NBD has an ATPase activity that controls the binding and release cycle of the substrate peptides (Rohland et al., 2022). A *dnaK* deletion mutant was constructed in *S. aureus* (Singh et al., 2012). The growth of this mutant was affected by heat and oxidative stress, while biofilm formation and adherence to A549 lung epithelial cells were impaired (Singh et al., 2012). Although DnaK is usually considered an intracellular protein, several extracellular functions of DnaK have been discovered (Rigo et al., 2020). DnaK was detected on the cell surface of *Mycoplasma hyorhinis*, and a DnaK recombinant protein interacted with human extracellular matrix proteins such as fibronectin, laminin, type IV collagen, and vitronectin in ELISAs (Li et al., 2022). Extracellular DnaK inhibits plasma alkaline phosphatase, which participates in pulmonary infection by *Francisella tularensis* (Arulanandam et al., 2012). In *Mycobacterium tuberculosis*, DnaK is found in secreted vesicles (Prados-Rosales et al., 2011), and extracellular DnaK polarizes macrophage phenotypes to an anti-inflammatory or immunosuppressive M2-like phenotype (Lopes et al., 2014).

We previously showed that CGRP activates the virulence of *S. epidermidis* MFP04, and that DnaK accumulates in the secretome of virulent CGRP-activated bacteria (N'Diaye et al., 2016). In this study, we explored a potential new role of *S. epidermidis* DnaK in biofilm formation in two skin commensal staphylococcal strains (*S. aureus* MFP03 and *S. epidermidis* MFP04) and in one clinical *S. aureus* strain (CIP 107093). *S. aureus* MFP03 and *S. epidermidis* MFP04 were previously isolated from healthy human skin (Hillion et al., 2013). The *S. aureus* CIP 107093 strain was supplied by the Biological Resource Center of the Pasteur Institute (Collection of the Pasteur Institute, Paris, France) and was previously defined as toxigenic due to its exfoliative toxin production, which is responsible for staphylococcal scalded skin syndrome (Becker et al., 1998). In this study, we investigated the potential role of extracellular *S. epidermidis* DnaK in the modulation of biofilm formation in both commensal and clinical staphylococcal strains, and assessed the contribution of its nucleotide-binding and substrate-binding domains. We also explored the impact of DnaK on the biofilm proteome of *S. aureus* CIP 107093.

## Materials and methods

### Sequence alignment

The DnaK sequence of *S. epidermidis* MFP04, a previously characterized CGRP-responsive strain, was compared with all available *S. epidermidis* DnaK sequences. The MFP04 protein shares more than 97% overall identity, with complete conservation of the regions containing the key amino acids targeted in this study. DnaK sequences (*Escherichia coli* K-12*, Staphylococcus aureus* MFP03, and *Staphylococcus epidermidis* MFP04) were obtained from the NCBI in FASTA format. The DnaK sequence of the *Staphylococcus aureus* CIP 107093 strain was provided by the Collection of Institut Pasteur (CIP) following the complete genome sequencing of the strain for this study. The sequences were aligned using the Clustal Omega multiple sequence alignment tool (https://www.ebi.ac.uk/Tools/msa/clustalo/). The ESPript 3.0 software (Robert and Gouet, 2014) (https://espript.ibcp.fr/ESPript/ESPript/) was used to visualize the data. Secondary structures were predicted based on the DnaK structure of *E. coli* K-12 (Protein Data Bank; PDB ID: 4B9Q; https://www.rcsb.org/structure/4B9Q).

### Bacterial strains and culture conditions

The strains used in this study and their origins are listed in Supplementary Table 2. Bacteria were grown overnight at 37 °C with shaking at 180 rpm. Tryptic Soy Broth (TSB) was used as the culture medium for all bacteria. Fresh medium supplemented with 0.5% glucose was inoculated (OD_580_ = 0.1) from overnight cultures for biofilm experiments. Growth curves were determined in 96-well microplates inoculated with bacterial suspensions in TSB supplemented with 0.5% glucose at an initial OD_580_ of 0.08. Purified rSep-DnaK protein or bovine serum albumin (BSA) (Sigma Aldrich) was added to each well at a final concentration of 1 μM. Cultures were grown at 37 °C under constant agitation. Growth curves were measured at OD_580_ every 15 min using a Bioscreen C automated microplate reader (Labsystems Oy, Helsinki, Finland) over at least three independent experiments.

### Whole-genome sequencing of the bacterial strain *Staphylococcus aureus* CIP 107093

Total DNA of CIP 107093 was extracted using the KingFisher Cell and Tissue DNA kit (Thermo Fisher Scientific) according to the manufacturer’s instructions. DNA quantification was performed using an ND-1000 spectrophotometer (NanoDrop Technologies), and DNA quality was assessed using a Qubit 3.0 Fluorometer (Invitrogen). Sequencing was carried out by the Mutualized Microbiology Platform (P2M) at the Institut Pasteur (Paris, France). DNA whole-genome shotgun sequencing libraries were prepared using the Nextera XT DNA library preparation kit (Illumina), and 2 × 150 bp paired-end sequencing was performed using an Illumina NextSeq 500 instrument. Sequence reads were trimmed and clipped using AlienTrimmer version 0.4.0 (Criscuolo and Brisse, 2013). Sequencing errors were corrected using Musket version 1.1 (Liu et al., 2013). *De novo* assembly was carried out using SPAdes version 3.12.0 (Bankevich et al., 2012). The draft genome sequence of strain CIP 107093 has a total size of 2,790,568 bp, represented in 73 scaffolds, with an N50 value of 79,530 bp.

The assembled genome was uploaded to NCBI under the accession number GCA_965117095 and annotated with the Prokaryotic Genome Annotation Pipeline.

### PCR amplification of the *dnaK* gene and plasmid construction

Chromosomal DNA from *S. epidermidis* MFP04 was purified using the Wizard® Genomic DNA Purification Kit (Promega) according to the manufacturer’s instructions. The *dnaK* gene was amplified with the Fwd-long-MFP04/Rev-long-MFP04 primers (Supplementary Table S3) using Phusion® Polymerase (NEB). The resulting *dnaK* amplicon was introduced into the pUC19 vector, which had been previously digested with SmaI (blunt-ended) (NEB), and ligated with T4 DNA ligase to generate pUC19-*dnaK*. The construct was verified by sequencing using the M13F/M13R primers (Supplementary Table S3). An NdeI site was inactivated in the coding sequence of *dnaK* using site-directed mutagenesis, without altering the encoded amino acid sequence. The oligonucleotide pair T210C-fwd/T210C-rev (Supplementary Table S3) and the plasmid pUC19-*dnaK* as the DNA template were used in PCR with Phusion© Polymerase (NEB). The resulting plasmid was then used as a template in a final PCR, with the oligonucleotides NdeI-*dnaK*/XhoI-*dnaK* primers (Supplementary Table S3). The PCR product was subsequently digested with NdeI and XhoI and ligated with plasmid pET22b+, which had been digested with the same restriction enzymes. The appropriate insert was confirmed by sequencing using T7 promoter and T7 terminator primers. Plasmids containing T173A and S397P mutations were constructed by GENEWIZ, using site-directed mutagenesis to replace ACA with GCA at nucleotide position 517, and TCT with CCT at nucleotide position 1,189, respectively.

### Heterologous expression and purification of rSep-DnaK

To overexpress the C-terminal His-tagged recombinant *S. epidermidis* DnaK proteins (rSep-DnaK-WT, rSep-DnaK-T173A, and rSep-DnaK-S397P), the following plasmids were transformed separately into *E. coli* BL21 (λDE3): pET22-*dnaK-WT*, pET22-*dnaK-T173A*, and pET22-*dnaK-S397P*. Transformed strains were subcultured from overnight cultures at 37 °C into 2 liters of TSB medium. Cells were grown at 37 °C with shaking at 180 rpm. Protein production was induced by the addition of 1 mM isopropyl-*β*-D-thiogalactopyranoside (IPTG, ThermoScientific) at an OD580 of 0.4 to 0.6. Cultures were incubated for an additional 2 h at 37 °C, and cells were harvested by centrifugation (15 min, 5,000 × g). Pellets were resuspended in lysis buffer (50 mM NaH_2_PO_4_, 300 mM NaCl, 10 mM imidazole, 1 mM ATP, 5 mM MgCl_2_, 2 mM 2-β-mercaptoethanol) and lysed by sonication (Branson 450 Digital Sonifier). Lysates were clarified by centrifugation for 1 h at 20,000 × *g*. Recombinant proteins were purified using Ni-NTA agarose (Qiagen), followed by gel filtration (HiPrep 16/60 Sephacryl S200, GE Healthcare) according to the manufacturer’s instructions. Proteins were recovered in phosphate-buffered saline (PBS) containing 1 mM ATP and 5 mM MgCl_2_. Purity and protein yield were confirmed by SDS-PAGE. Proteins were stored at 4 °C. The concentration of the recombinant protein variants was determined using the Pierce BCA Protein Assay Kit (ThermoFisher).

### Crystal violet biofilm assay

Overnight cultures of strains *S. aureus* MFP03, *S. epidermidis* MFP04 and *S. aureus* CIP 107093 were diluted to an OD_580_ of 0.1 in TSB containing 0.5% glucose. Purified rSep-DnaK or BSA (Sigma Aldrich) was added to each well at a final concentration of 1 μM. This concentration was selected based on preliminary optimization assays showing that it was the lowest concentration that affected biofilm formation reproducibly without impacting bacterial growth. The plates were incubated at 37 °C for 24 h. Ninety-six-well flat-bottom polystyrene plates (Corning) were used. The supernatant from each well was removed, and biofilms were washed with 200 μL of PBS. Two hundred μl of 0.1% crystal violet solution was added to each well, and the plate was incubated at room temperature for 15 min to stain the adherent biofilm. Crystal violet was removed, and the biofilms were washed with 200 μL of PBS. To solubilize the remaining crystal violet, 200 μL of 30% acetic acid was added to each well. Absorbance was measured at OD_595_ using a Tecan Spark© reader. BSA-treated wells were used as a control, with absorbance set to 100%. Each data point was the average of 10 replicate wells. Error bars represent the standard error of the mean (SEM). The results were analyzed with a paired or unpaired *t*-test and are representative of three independent experiments. Growth curves performed for each strain in the presence or absence of rSep-DnaK confirmed that biofilm variations were not due to growth defects (Supplementary Figure S2).

### Confocal laser scanning microscopy

For biofilm imaging using confocal laser scanning microscopy (CLSM), overnight cultures of *S. aureus* MFP03, *S. epidermidis* MFP04 and *S. aureus* CIP 107093 were diluted to an OD_580_ of 0.1 in TSB containing 0.5% glucose. Purified rSep-DnaK or BSA was added to a final concentration of 1 μM, and the cultures were incubated at 37 °C for 24 h. Twenty-four-well glass-bottom plates (Cellvis) were used. Biofilms were washed with PBS to remove non-adherent cells. Bacterial biofilms were stained with 5 × 10^−3^ M SYTO9 green-fluorescent dye (Invitrogen). eDNA was stained with 1 × 10^−6^ M DDAO (Invitrogen). Proteins were stained with 1 × SyproRuby (ThermoFisher). The *β*1-3 and *β*1-4 polysaccharides were stained with CalcoFluor White M2R at a concentration of 200 μg/mL (Sigma-Aldrich). CLSM imaging of biofilms was performed using a Zeiss LSM710 microscope (Carl Zeiss microscopy) with a 63 × oil immersion objective. Images were taken every micrometer through the entire biofilm depth. For visualization and processing of 3D images, Zen 2.1 SP1 software (Carl Zeiss microscopy) was used. Biofilm average thickness (μm), biovolume (μm^3^/μm^2^), and roughness coefficient were determined using COMSTAT2 software (Heydorn et al., 2000) (http://www.imageanalysis.dk/). eDNA, protein, and polysaccharide values were normalized to the biofilm biovolume. Five to ten image stacks from three independent experiments were used for each analysis.

### Proteolytic activity assay

*Staphylococcus aureus* strains MFP03 and CIP 107093, and *Staphylococcus epidermidis* MFP04 were evaluated for proteolytic activity using skim milk agar plates. A modified tryptic soy agar medium was made by adding 10% skim milk to the original tryptic soy agar (Sigma-Aldrich, Merck Group, Saint-Quentin-Fallavier, France). Bacterial strains were grown overnight in tryptic soy broth (TSB) at 37 °C with shaking, and cultures were adjusted to an optical density at 600 nm (OD₆₀₀) of 2.0. Five-microliter aliquots of each suspension were spotted in quintuplicate onto the surface of skim milk TSA plates and incubated at 25 °C for 72 h. Proteolytic activity was determined by the formation of clear zones surrounding the colonies, indicative of casein degradation. The diameter of the lysis halos was measured to provide a semi-quantitative estimate of protease activity. The assay was performed in three independent biological replicates, and the figure presented is representative of the results obtained across all replicates.

### Microscale thermophoresis

The rSep-DnaK protein was labeled using the Red Tris-NTA 2nd gen. Labeling kit (Cat MO-L018, NanoTemper Technologies GmbH). The labeling reaction was performed according to the manufacturer’s instructions in PBS with the addition of 1 mM ATP, using a 20 × 10^−9^ M protein concentration at room temperature for 30 min. Peptide NRLLLTG (Sigma Aldrich) was dissolved in MST buffer supplemented with 0.01% Tween-20. A series of 1:1 dilutions was prepared in the same buffer, resulting in ligand concentrations ranging from 4.6 × 10^−9^ M to 150 × 10^−6^ M. For the microscale thermophoresis (MST) experiment, each ligand dilution was mixed with an equal volume of rSep-DnaK, resulting in a final concentration of rSep-DnaK of 10 × 10^−9^ M and final ligand concentrations at half of the aforementioned ranges. Instrument parameters were set to 50% light-emitting diode power and medium MST power. Data from three independent measurements were analyzed (NT. Analysis software version 1.5.41, NanoTemper Technologies GmbH). The Microscale Thermophoresis (MST) signal was expressed as F_norm_, calculated by normalizing the fluorescence recorded prior to the temperature gradient (F_cold_) to that measured between 14 and 15 s (F_hot_), as described previously (Cox and Mann, 2008). The resulting dose–response curves were displayed as the mean value (± SD) obtained from n = 3, and fitted using the manufacturer’s implementation of the law of mass action (K_d_ model) to determine the dissociation constant (K_d_).

### Total protein extraction

An overnight culture of *S. aureus* CIP 107093 was diluted to an optical density of 0.1 at 580 nm in TSB. Biofilms were grown in 24-well plates and incubated at 37 °C for 24 h with or without 1 μM rSep-DnaK. Following incubation, biofilms were harvested and lysed using a Precellys homogenizer (Bertin Technologies) at 5,000 rpm for 5 min at 4 °C. Lysates were centrifuged at 20,000 × g for 20 min at 4 °C, and supernatants were collected. Proteins were precipitated by the addition of 20% (w/v) trichloroacetic acid (TCA) and incubated overnight at 4 °C with gentle agitation (50 rpm). Precipitated proteins were recovered by centrifugation at 20,000 × g for 30 min at 4 °C, washed three times with cold acetone, and air-dried prior to downstream analysis.

### Protein sample preparation

Protein pellets were solubilized by adding 4% SDS and 50 mM Tris–HCl, pH 7.5, and then homogenized on a Covaris E220 sonicator using Adaptive Focused Acoustics (AFA) technology. Samples were collected into m-130 glass fiber screw cap tubes and sonicated using 175 peak incident power (PIP), 200 cycles per burst (CPB), and a 10% duty factor for 180 s at 20 °C. Protein samples were clarified by centrifugation at 20,000 × g at room temperature for 5 min, and the protein-containing supernatants were transferred to clean tubes. Protein disulfide bonds were reduced using 10 mM TCEP for 30 min at room temperature. Alkylation of the reduced disulfide bridges was performed using 20 mM iodoacetamide for 30 min at room temperature in the dark. Protein samples were then processed according to the SP3 protocol (Hughes et al., 2019). Sera-Mag Carboxylate Modified SpeedBeads (45,152,105,050,250 and 65,152,105,050,250) from Cytiva were combined in a 1:1 (v/v) ratio, washed three times with water, and resuspended in water at a final concentration of 50 μg/μL. Magnetic beads were added to the protein samples at a 10:1 (w/w) beads-to-protein ratio. Protein capture on the magnetic beads was induced by adding acetonitrile in a 7:1 (v/v) acetonitrile-to-protein ratio. Sample mixtures were incubated on a ThermoMixer at 1,000 rpm for 5 min at room temperature. Sample mixtures were placed in a magnetic rack and incubated at room temperature until the beads migrated to the tube wall. Magnetic beads with bound proteins were pulled to the side of the microcentrifuge tubes, and the supernatants were removed as waste. Magnetic beads with bound proteins were washed three times with 80% acetonitrile in water (v/v) off the magnet. The magnet was placed back on the rack, and sample mixtures were incubated until the beads migrated to the tube wall. The contaminant-containing supernatants were removed as waste. Sequencing Grade Modified Trypsin was added at a 1:8 (w/w) enzyme-to-protein ratio with 100 mM TEAB. Protein samples were digested on a ThermoMixer at 37 °C and 1,000 rpm overnight. Peptide samples were then centrifuged at 20,000 × g at room temperature for 1 min. Sample tubes were placed on the magnetic rack and incubated until bead pellets migrated to the tube wall. The peptide-containing supernatants were collected into clean sample vials. Peptide samples were dried and stored at −80 °C without additional cleanup until further use. Peptides were resuspended in 0.1% FA prior to LC–MS injection.

### Liquid chromatography-mass spectrometry

LC–MS/MS analysis was performed on an Orbitrap Q Exactive Plus Mass Spectrometer (Thermo Fisher Scientific) coupled with a Proxeon EASY-nLC 1,200 (Thermo Fisher Scientific). One μg of peptides was injected onto a PepMap RSLC C_18_ 50 cm column (2 μm particle size, 100 Å pore size). Column equilibration and peptide loading were conducted at 900 bars in buffer A (0.1% FA). Peptides were separated using a multi-step gradient: from 2 to 7% buffer B (80% ACN, 0.1% FA) over 5 min, from 7 to 23% buffer B over 70 min, from 23 to 45% buffer B over 30 min, and from 45 to 95% buffer B over 5 min, at a flow rate of 250 nL/min. The column temperature was set to 60 °C. MS data were acquired using Xcalibur software with a data-dependent method. MS scans were obtained at a resolution of 70,000, and MS/MS scans (fixed first mass 100 m/z) at a resolution of 17,500. The AGC target and maximum injection time for the survey scans and the MS/MS scans were set to 3E^6^, 20 ms, and 1E^6^, 60 ms, respectively. An automatic selection of the 10 most intense precursor ions was activated (Top 10) with a 45 s dynamic exclusion. The isolation window was set to 1.6 m/z, and the normalized collision energy was fixed at 28 for HCD fragmentation. We used an underfill ratio of 1.0% corresponding to an intensity threshold of 1.7E^5^. Unassigned precursor ion charge states, as well as 1, 7, 8, and >8 charged states, were excluded, and the peptide match feature was disabled.

### Proteomic data analysis

Acquired raw files were analyzed using MaxQuant software version 2.1.4.0 (Cox et al., 2011) using the Andromeda search engine (Cox and Mann, 2008; Tyanova et al., 2016). Samples were grouped by the type of experiment. The MS/MS spectra were searched against the *S. aureus* MFP03 (2,504 entries), *S. epidermidis* MFP04 (2,269 entries), and *S. aureus* CIP 107093 (2,634 entries).

All searches were performed with oxidation of methionine and protein N-terminal acetylation as variable modifications and cysteine carbamidomethylation as a fixed modification. Trypsin was selected as the protease, allowing for up to two missed cleavages. The minimum peptide length was set to five amino acids, and the peptide mass was limited to a maximum of 8,000 Da. The false discovery rate (FDR) for peptide and protein identification was set to 0.01. The main search peptide tolerance was set to 4.5 ppm, and the MS/MS match tolerance was set to 20 ppm. Second peptides were enabled to identify co-fragmentation events. A unique peptide to the protein group was required for protein identification. A false discovery rate cut-off of 1% was applied at the peptide and protein levels. The mass spectrometry proteomics data have been deposited into the ProteomeXchange Consortium via the PRIDE partner repository with the dataset identifier PXD062742.

### Pairwise comparison

Protein intensities were normalized by condition using the median-centering function from the DAPAR R package (Wieczorek et al., 2017), and missing values were imputed using the impute.mle function from the imp4p R package (Gianetto et al., 2020). This algorithm imputes values in a condition only when an intensity value has been quantified in at least one of the samples from that condition. To determine if a protein is significantly differentially abundant between the two conditions, a moderated t-test was performed using the limma R package (Ritchie et al., 2015). Moreover, peptides quantified in one condition but not in the other were also considered differentially abundant. An adaptive Benjamini-Hochberg procedure was applied to the resulting *p*-values using the adjust.p function from the R package cp4p (Giai Gianetto et al., 2016) and a false discovery rate (FDR) threshold of 5% was used to select proteins that evolved differently between the two conditions.

### Functional analysis

Quantitative proteomic data were analyzed using Proteomaps and visualized as Voronoi treemaps (Liebermeister et al., 2014). To enable the integration of CIP 107093 protein data into the Proteomaps server, proteins that were more abundant in one condition compared to the other, including those detected exclusively in one condition, were BLASTed against the *Staphylococcus aureus* COL and N315 reference proteomes. Only the top match with a percent identity above 30% was retained for each sequence. Proteins that either had no significant match or shared an identifier with another matched protein from the same strain were manually added to the Proteomaps input database. This customized database was then submitted to the server for treemap generation.

In the final Proteomaps, an asterisk indicates proteins uniquely detected in one condition but absent in the other; for visualization purposes, their log₂(fold change) values were arbitrarily set to 10.

### Structure prediction using AlphaFold3

The three-dimensional structure of *S. epidermidis* DnaK was predicted using AlphaFold3 (Abramson et al., 2024) (https://alphafoldserver.com). The protein sequence used for the prediction is provided in Supplementary Figure S3. The highest-confidence model was selected for subsequent analyses and visualized with PyMOL. RMSD values were calculated on aligned Cα atoms to quantify the structural similarity between the two DnaK proteins. The alignment statistics are summarized in Supplementary Table S1.

### Statistical analysis

Experiments were performed independently at least three times. The normality of the data was assessed using the Shapiro–Wilk test. Comparisons between two groups were performed using either Student’s *t*-test or paired *t*-test, as appropriate. For experiments with multiple groups, one-way ANOVA was used after confirming homogeneity of variances with the Brown–Forsythe test. When variances were unequal, Welch’s ANOVA followed by Dunnett’s T3 *post hoc* test was applied; otherwise, Dunnett’s or Tukey’s post hoc tests were used for multiple comparisons. Statistical significance was defined as *p* < 0.05. All analyses were conducted using GraphPad Prism 10.6.1.

## Results

### Recombinant rSep-DnaK protein reduces biofilm formation in *S. aureus* CIP 107093

**Figure 1:**
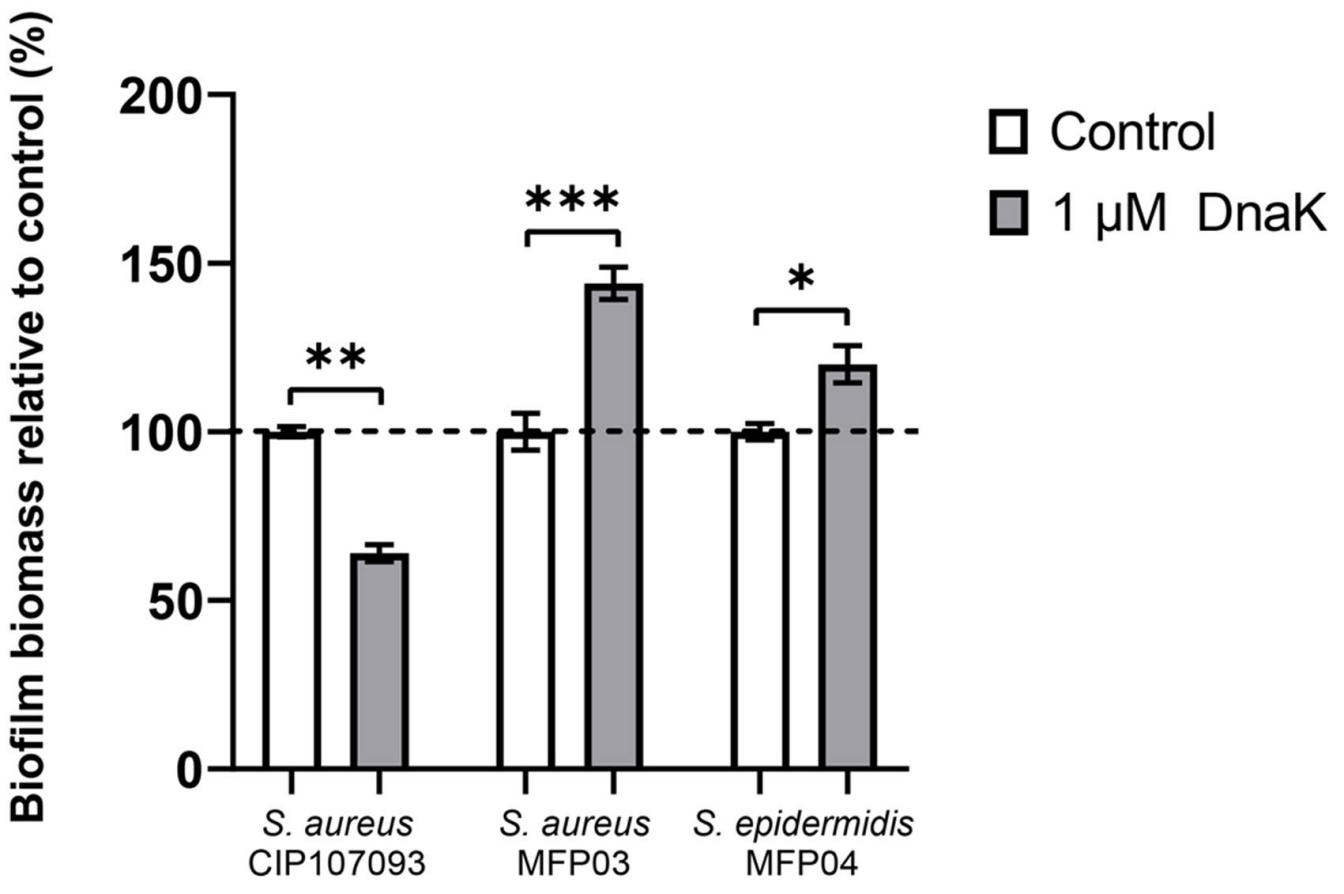
Relative biofilm formation of *Staphylococcus aureus* CIP 107093, *Staphylococcus aureus* MFP03, and *Staphylococcus epidermidis* MFP04 cultures treated with rSep-DnaK protein or BSA. Biofilms were cultured in TSB supplemented with 0.5% glucose at 37 °C for 24 h and treated with either rSep-DnaK protein or BSA (set as 100% control). Data are presented as mean values (± SEM) and are representative of three independent experiments. Statistical significance was determined using paired t-tests, with the following *p*-values: *p < 0.05; ***p < 0.001.

We initially expressed and purified the recombinant DnaK chaperone protein from the *Staphylococcus epidermidis* MFP04 strain, designated rSep-DnaK (Supplementary Figure S1). For further experimental details, refer to the Materials and Methods section. We next focused on the impact of rSep-DnaK on the growth and biofilm formation of *S. aureus* CIP 107093, *S. aureus* MFP03, and *S. epidermidis* MFP04. As shown in Supplementary Figure S2, rSep-DnaK had no effect on the growth of any of the three strains. To assess the role of DnaK in biofilm formation, 1 μM purified rSep-DnaK was added to the cultures at the beginning of biofilm development. Biofilm formation was quantified by crystal violet staining. As shown in Figure 1, *S. aureus* CIP 107093 treated with rSep-DnaK formed 35% less biofilm mass than the BSA-treated biofilm, which was used as a control since BSA had no effect on biofilm formation in any of the tested strains. Conversely, in both *S. aureus* MFP03 and *S. epidermidis* MFP04 strains, the addition of rSep-DnaK increased biofilm formation by up to 144 and 120%, respectively. Thus, rSep-DnaK reduces the biofilm formation of the clinical strain (*S. aureus* CIP 107093), while it promotes biofilm formation in two commensal strains isolated from healthy skin (*S. aureus* MFP03 and *S. epidermidis* MFP04).

**Figure 2:**
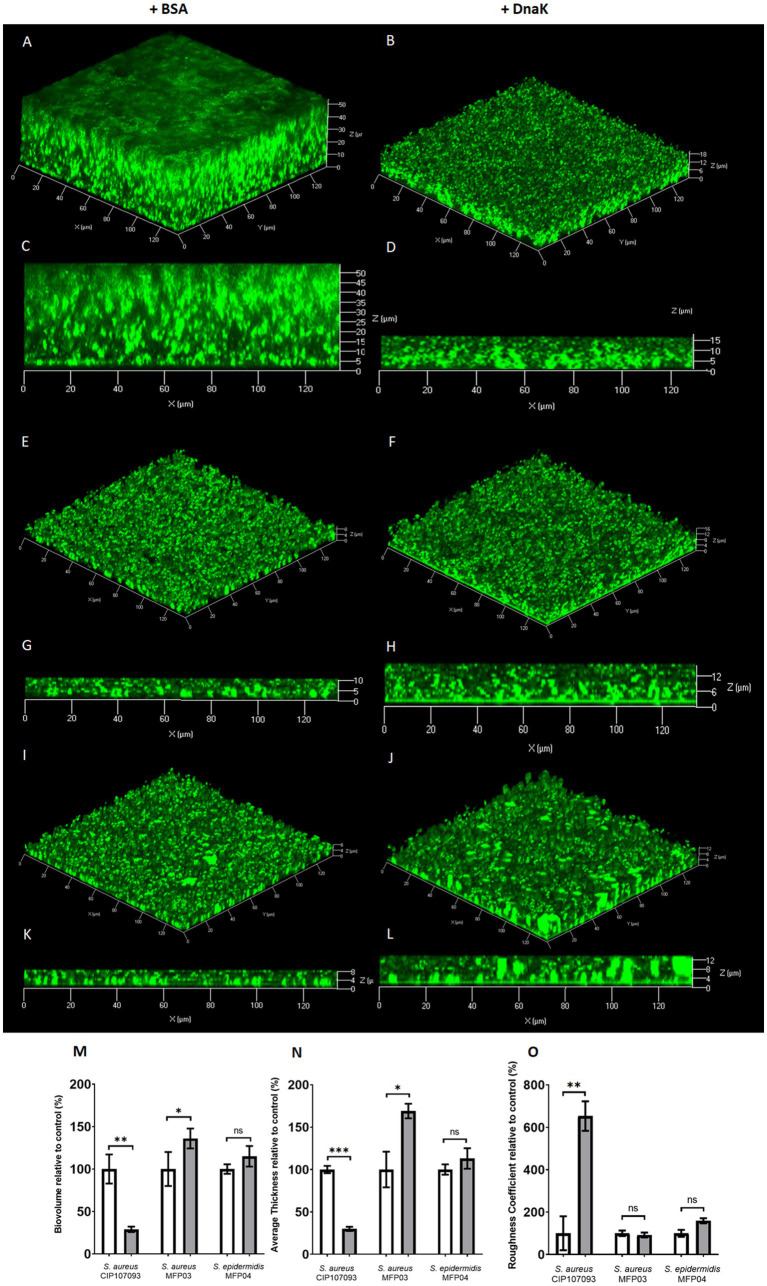
Modification of biofilm architecture by rSep-DnaK in *Staphylococcus aureus* CIP 107093, *Staphylococcus aureus* MFP03, and *Staphylococcus epidermidis* MFP04. Bacterial cells within biofilms were stained green using SYTO9. Image acquisition was performed using CLSM. 3D visualization of the biofilm and sectional views were generated for the strains *Staphylococcus aureus* CIP 107093 **(A–D)**, *Staphylococcus aureus* MFP03 **(E–H)**, and *Staphylococcus epidermidis* MFP04 **(I–L)**. Biofilms were treated with either rSep-DnaK protein or BSA (set as 100% control). The biofilm biovolume **(M)**, average thickness **(N)**, and roughness coefficient **(O)** were quantified using COMSTAT 2.1. The results, presented as mean values (± SEM), are representative of three independent experiments. Statistical significance was determined using paired *t-t*ests, with the following *p*-values: *p < 0.05; **p < 0.01; ***p < 0.001.

To characterize biofilms formed in the presence of the rSep-DnaK protein (Figures 2A–L), we quantified biofilm parameters using confocal laser scanning microscopy (CLSM), specifically biofilm biovolume (Figure 2M), average thickness (Figure 2N), and roughness coefficient (Figure 2O). For strain *S. aureus* CIP 107093, we observed a 70% decrease in biofilm biovolume (Figure 2M) compared to the control. For *S. aureus* strain MFP03, the addition of rSep-DnaK increased biovolume by up to 176%, while no significant difference was observed for *S. epidermidis* MFP04. The average thickness (Figure 2N) of the *S. aureus* CIP 107093 biofilm treated with rSep-DnaK also decreased by 70%. For *S. aureus* MFP03, average biofilm thickness increased by 69%, whereas no significant difference was noted for the *S. epidermidis* MFP04 biofilm. As shown in Figure 2O, the roughness coefficient increased by a factor of 7.5 for rSep-DnaK-treated *S. aureus* CIP 107093, indicating a rougher biofilm surface. However, no significant difference in roughness was observed for *S. aureus* MFP03 or *S. epidermidis* MFP04.

### S397P mutation in the substrate-binding domain of *Staphylococcus epidermidis* DnaK disrupts peptide binding

**Figure 3:**
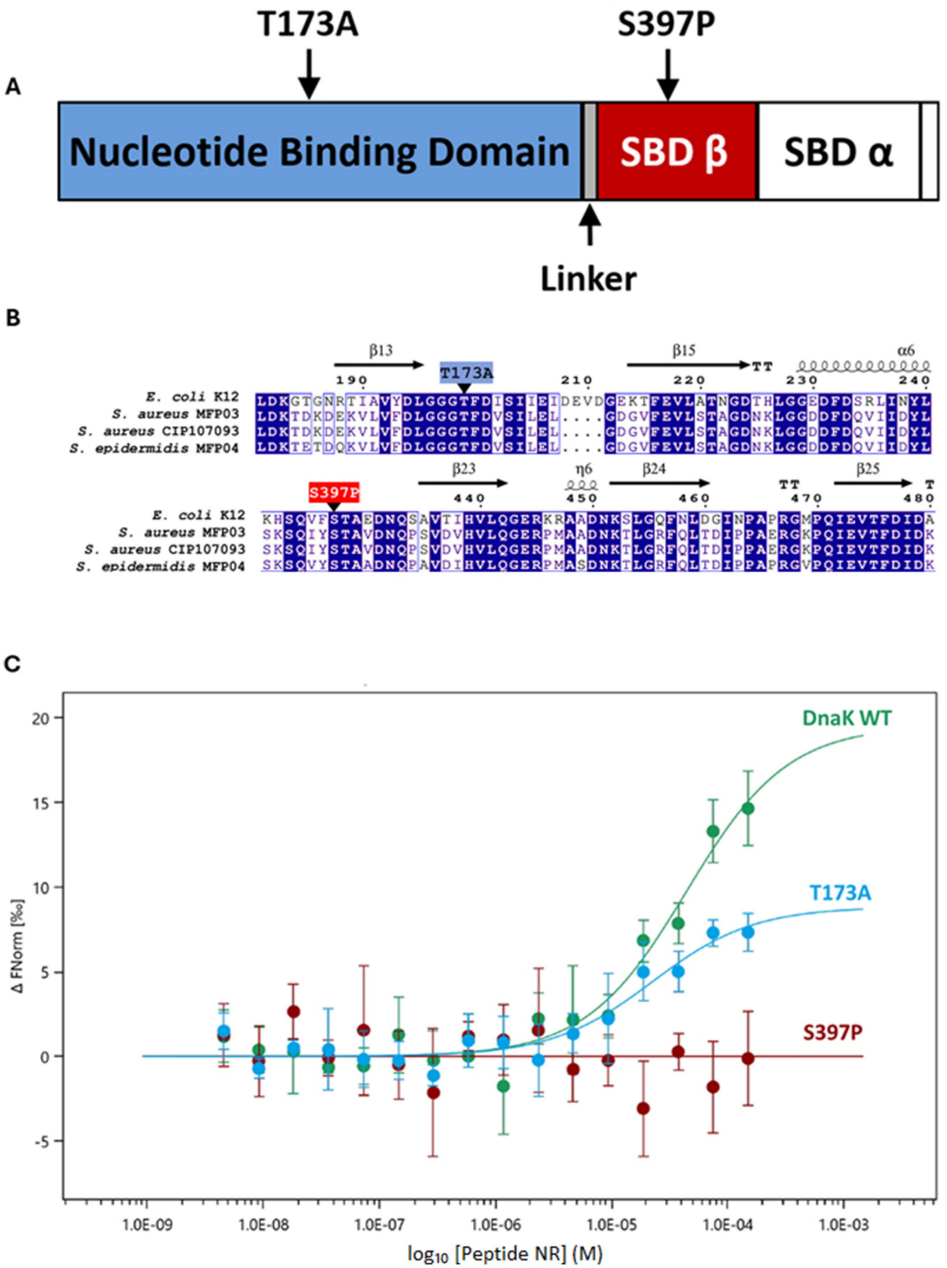
*In vitro* characterization of rSep-DnaK proteins with targeted point mutations. **(A)** Protein domain architecture of rSep-DnaK. The protein is composed of an N-terminal nucleotide-binding domain (NBD), a linker, and a substrate-binding domain, which is divided into two subdomains (SBD-*α* and SBD-*β*) in the C-terminal region. The mutations in the *Staphylococcus epidermidis* DnaK sequence used in this study are indicated by arrows. **(B)** Primary amino acid sequence alignments of DnaK from *Escherichia coli* K12, *Staphylococcus aureus* MFP03, *Staphylococcus aureus* CIP 107093, and *Staphylococcus epidermidis* MFP04. The secondary structures of *E. coli* are displayed above the alignment (helices, β-strands shown as arrows, and turns indicated by TT). Identical residues and highly conserved residues (>70% similarity) are highlighted with a blue background or in blue text, respectively. The residues highlighted in blue and red correspond to the mutations in the *Staphylococcus epidermidis* DnaK sequence analyzed in this study. The figure was generated using the ESPript server. **(C)** Direct affinity measurement of purified rSep-DnaK for the NRLLLTG peptide using microscale thermophoresis. The WT, T173A, and S397P rSep-DnaK proteins were fluorescently labeled and incubated with varying concentrations of the peptide NRLLLTG, and direct interaction was quantified. The data are representative of three independent experiments.

Multiple sequence alignments using DnaK primary sequences from *E. coli* K-12, *S. aureus* MFP03, and *S. epidermidis* MFP04 reveal a high degree of conservation (Supplementary Figure S3). The DnaK protein of *E. coli* shares 58% sequence identity with the DnaK proteins of the three staphylococcal strains used in this study, whereas the primary sequences of DnaK from *S. epidermidis* MFP04 and either *S. aureus* MFP03 or *S. aureus* CIP 107093 share 90% sequence identity. In *E. coli*, residue T199 lies within the nucleotide-binding domain of DnaK, and S427 lies within the substrate-binding domain-*β* (Figures 3A,B). The substitution mutation T199A abolishes ATPase activity in *E. coli* (Qi et al., 2013), whereas the S427P substitution affects peptide binding affinity (Chiappori et al., 2015; Montgomery et al., 1999). Circular dichroism analyses revealed that the secondary structure of the S427P mutant is highly similar to that of the wild-type DnaK protein (Montgomery et al., 1999). Based on the alignment results, we generated two corresponding *S. epidermidis* DnaK mutants: T173A and S397P. The resulting recombinant *S. epidermidis* proteins rSep-DnaK-WT, rSep-DnaK-T173A, and rSep-DnaK-S397P were produced and purified in *E. coli* BL21 λDE3 (Supplementary Figure S1). To assess the structural relevance of these substitutions in the staphylococcal ortholog, we generated a full-length AlphaFold model of *S. epidermidis* DnaK (AF3), which received a high score of 0.83 for its predicted template modeling (pTM) and a mean predicted local-distance difference test (pLDDT) score exceeding 90, reflecting a very high degree of accuracy. Structural alignment with the crystallized *E. coli* DnaK structure (PDB ID: 4B9Q) revealed a high degree of conservation, particularly within the nucleotide-binding domain (NBD) and the *β*-subdomain of the substrate-binding domain (SBD-β), where the two substituted residues, T173 and S397, are located, respectively (Supplementary Figure S4; Supplementary Table S1). These observations support the hypothesis that the functional consequences of these mutations in *S. epidermidis* are likely to mirror those previously described in *E. coli*. In the AlphaFold model, the S397P mutation, introduced into the *S. epidermidis* sequence, maps to a surface-exposed loop in the SBD-β. While no significant structural rearrangement was revealed, the mutation appears to rigidify the loop locally and extend the adjacent β-strand by one residue. In contrast, the *α*-subdomain of the SBD (SBD-α) in the C-terminal region of *S. epidermidis* DnaK revealed minor differences compared to *E. coli* DnaK, including four additional α-helical turns followed by a disordered segment. The peptide NRLLLTG (NR) has been extensively characterized for its high affinity for DnaK (Gragerov et al., 1994). This peptide was selected as a model substrate to investigate its interactions with the protein, focusing on the wild-type protein as well as on mutants in the substrate-binding domain (SBD) and nucleotide-binding domain (NBD), thereby allowing precise assessment of the effects of mutations in the SBD and NBD on the binding affinities of rSep-DnaK. For the first time, an interaction equivalent to that observed in *E. coli* was detected in *S. epidermidis*, as rSep-DnaK-WT bound to the NRLLLTG peptide (Kd = 44.4 μM; Figure 3C). The rSep-DnaK-S397P mutant showed no binding, highlighting the critical impact of the S397P mutation on peptide interaction in *S. epidermidis*, consistent with the results observed in *E. coli*. Additionally, although the rSep-DnaK-T173A mutant is expected to lack ATPase activity, it retained a binding affinity similar to that of the wild-type protein (Kd = 22.5 μM; Figure 3C), indicating that ATP hydrolysis may be dispensable for NRLLLTG binding, in line with observations made for the *E. coli* T199A mutant (Barthel et al., 2001).

### Critical role of the substrate-binding domain of *Staphylococcus epidermidis* DnaK for biofilm inhibition in *Staphylococcus aureus* CIP 107093

**Figure 4:**
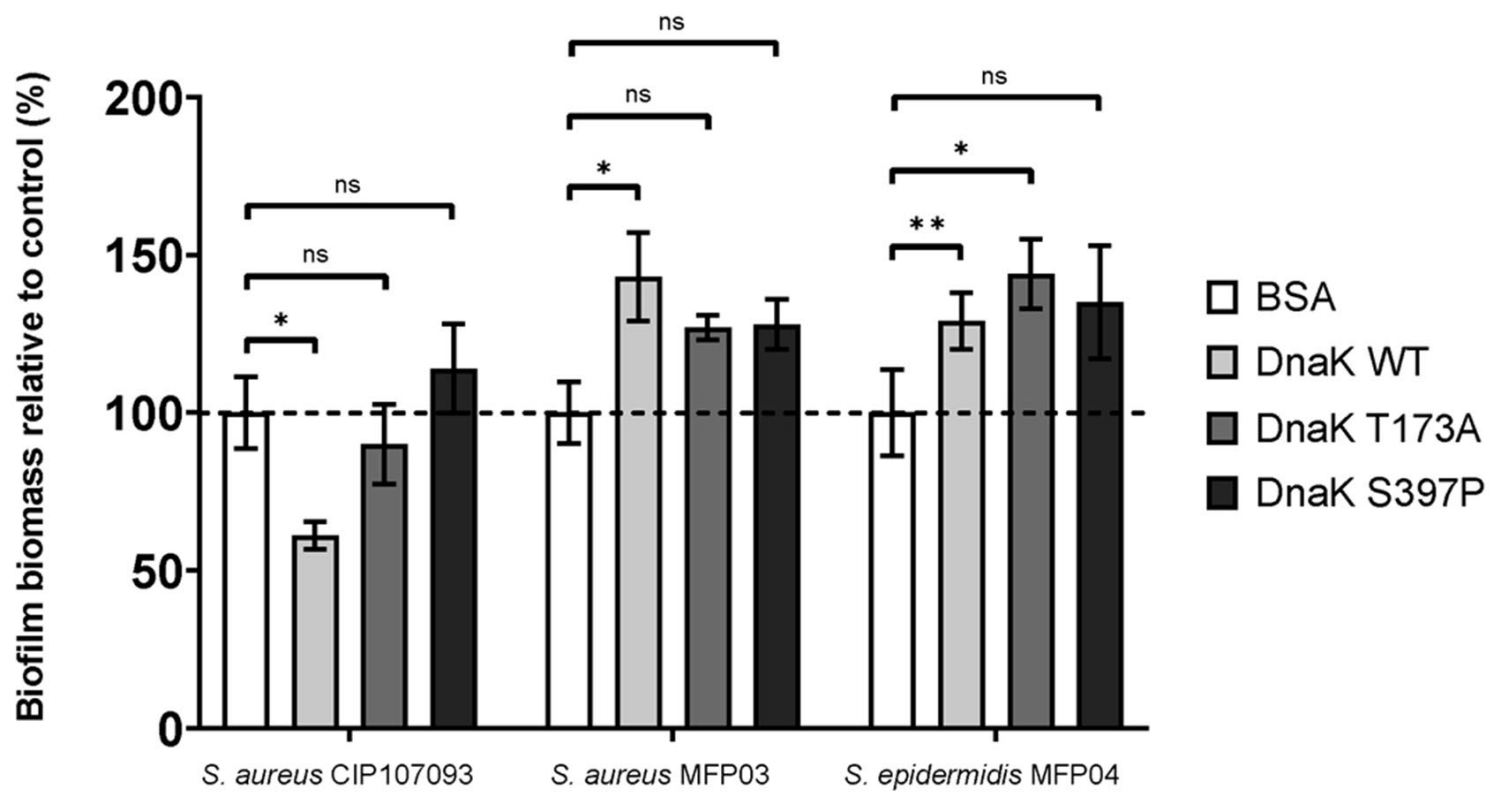
The critical roles of S397 (SBD) and T173 (NBD) residues of rSep-DnaK in biofilm inhibition in *Staphylococcus aureus* CIP 107093. Biofilm formation of *Staphylococcus aureus* CIP 107093, *Staphylococcus aureus* MFP03, and *Staphylococcus epidermidis* MFP04 following treatment with rSep-DnaK-WT, rSep-DnaK-T173A, rSep-DnaK-S397P, or BSA (protein control, set as 100%). Data are presented as mean values (± SEM), representing three independent experiments. Statistical significance was determined by one-way ANOVA with Dunnett’s *post hoc* test for comparisons against the control group (BSA), with *p*-values: *p < 0.05; **p < 0.01.

To understand the molecular mechanisms underlying the effect of extracellular DnaK protein on biofilm formation, we produced two rSep-DnaK proteins carrying amino acid substitutions T173A and S397P in the NBD and SBD, respectively (Figures 3A,B). Each purified protein was added at the beginning of biofilm development, and biofilm formation was quantified by crystal violet staining. In *S. aureus* CIP 107093, the rSep-DnaK-WT protein reduced biofilm formation by 40%, whereas neither the rSep-DnaK-S397P protein nor the rSep-DnaK-T173A protein led to a significant reduction (Figure 4). In the commensal strains *S. aureus* MFP03 and *S. epidermidis* MFP04, biofilm formation was not reduced by the rSep-DnaK-WT protein, as previously observed, and showed similar levels with the rSep-DnaK-S397P and rSep-DnaK-T173A proteins. Given the allosteric coupling between the NBD and SBD regions, these results indicate that DnaK’s ability to inhibit biofilm formation in *S. aureus* CIP 107093 may depend on functional substrate binding, reflecting conformational changes that alter chaperone activity. Whether this effect relies on a basal ATPase activity independent of cochaperones, as previously described for DnaK, or instead involves cochaperones such as DnaJ, remains to be clarified.

To confirm the involvement of the substrate-binding domain (SBD) in biofilm inhibition, we employed the NR peptide, a synthetic ligand designed to selectively target the SBD of *E. coli* DnaK (Gragerov et al., 1994; Stevens et al., 2003). As CGRP was previously shown to have no impact on biofilm formation in *S. aureus* MFP03 (N'Diaye et al., 2016) and did not affect biofilm formation in *S. aureus* CIP 107093 (data not shown), it was chosen as a negative control. Each peptide was introduced at a concentration of 1 μM at the onset of biofilm development, in the presence or absence of 1 μM *S. epidermidis* rSep-DnaK-WT. While CGRP had no detectable effect on DnaK-mediated biofilm inhibition, NR fully abrogated this effect, restoring biofilm formation to levels comparable to the BSA control (Supplementary Figure S5).

Collectively, these findings establish the SBD of *S. epidermidis* DnaK as a key determinant in the suppression of biofilm formation in *S. aureus* CIP 107093.

### Distinct biofilm composition and elevated proteolytic activity in *Staphylococcus aureus* CIP 107093

**Figure 5:**
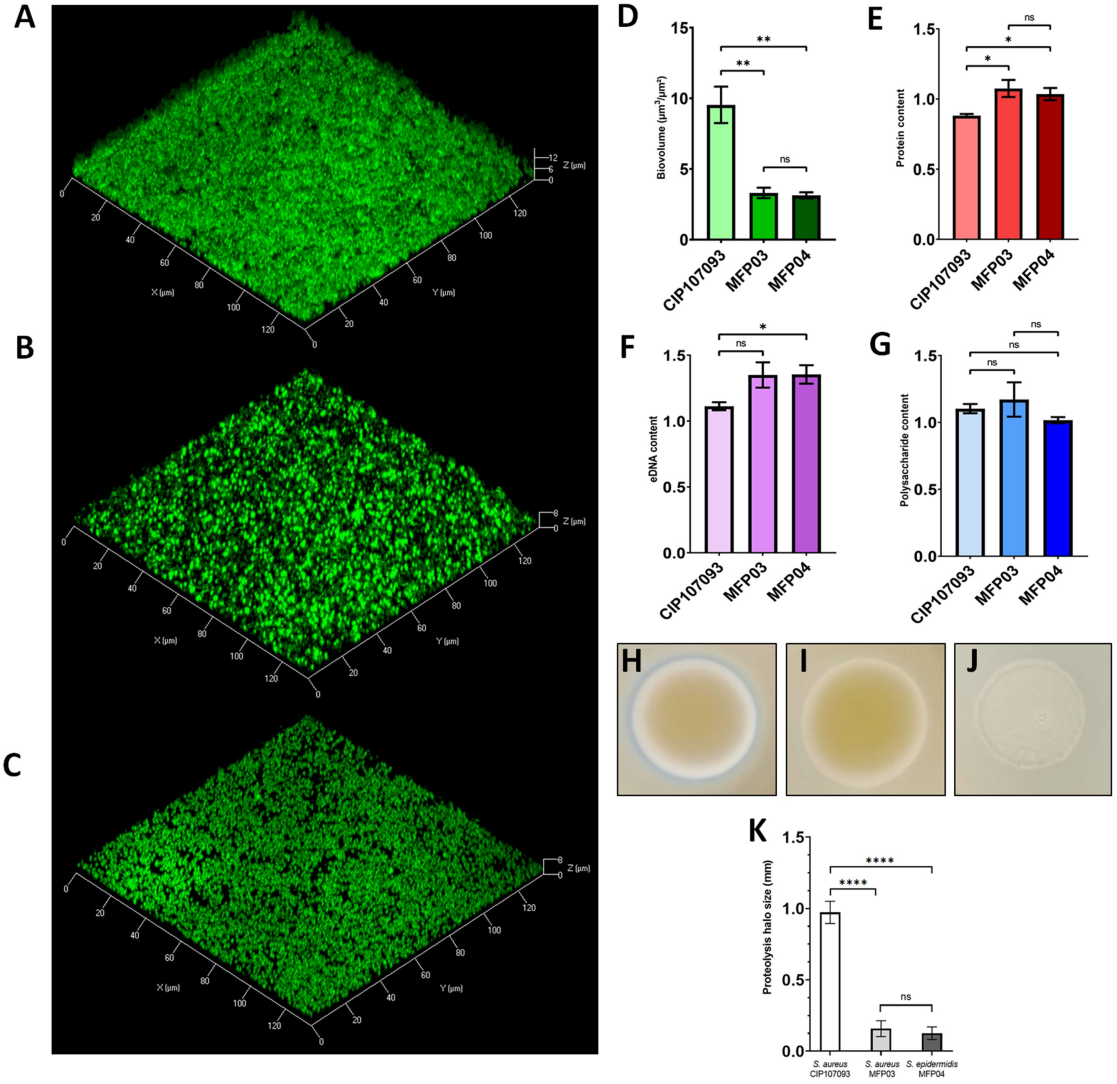
Biofilm composition and proteolytic activity of *Staphylococcus aureus* CIP 107093, *Staphylococcus aureus* MFP03, and *Staphylococcus epidermidis* MFP04. Bacterial cells within biofilms were stained green using SYTO9. Proteins were stained red with SyproRuby. Extracellular DNA (eDNA) was stained red with DDAO. β1-3 and β1-4 polysaccharides were stained blue using CalcoFluor White (CFW). Images were acquired by confocal laser scanning microscopy (CLSM) and are presented as 3D shadow representations of *Staphylococcus aureus* CIP 107093 **(A)**, *Staphylococcus aureus* MFP03 **(B)**, and *Staphylococcus epidermidis* MFP04 **(C)** biofilm structures. Biofilms were grown under static conditions at 37 °C for 24 h. Bacterial biovolume **(D)**, proteins **(E)**, eDNA **(F)**, and β1-3/β1-4 polysaccharides **(G)** are expressed as volumetric density (μm^3^/μm^2^), normalized to total biofilm biovolume. The results presented as mean values (± SEM) are representative of three independent experiments. Proteolytic activity was assessed in *Staphylococcus aureus* CIP 107093 **(H)**, *Staphylococcus aureus* MFP03 **(I)**, and *Staphylococcus epidermidis* MFP04 **(J)** by normalizing culture density in TSB and incubating on skim-milk agar at 25 °C for 72 h. Proteolytic activity **(K)** was quantified based on the diameter of the proteolysis halo in *Staphylococcus aureus* CIP 107093 (white), *Staphylococcus aureus* MFP03 (light gray), and *Staphylococcus epidermidis* MFP04 (dark gray). Halos were measured in pixels using Photoshop CS6 Portable and converted to millimeters. Data represent the mean of at least 9 measurements across three biological replicates (± SEM). Statistical significance was determined by Welch’s ANOVA with Dunnett’s T3 post hoc test for panels (D–G), and one-way ANOVA with Tukey’s multiple comparison test for panel (K) with *p*-values: *p < 0.05; **p < 0.01; ****p < 0.0001.

As shown in Figures 1, 2, the rSep-DnaK protein affects biofilm formation differently across the three tested staphylococcal strains. To better understand the differential impact of recombinant DnaK on biofilm formation among the three staphylococcal strains, we first characterized the basal composition of biofilms in the absence of this protein. We quantified the relative abundance of proteins, extracellular DNA (eDNA), and polysaccharides in each biofilm matrix, normalizing to biofilm biovolume. Biofilm cells, proteins, eDNA, and polysaccharides were stained with SYTO9, SyproRuby, DDAO, and CFW fluorescent dyes, respectively. Confocal laser scanning microscopy (CLSM) images were acquired and analyzed using COMSTAT 2.1 software. Figures 5A–C show fluorescence micrographs of *S. aureus* CIP 107093, *S. aureus* MFP03, and *S. epidermidis* MFP04 biofilms. *S. aureus* CIP 107093 biofilms exhibited a higher biovolume compared to those formed by *S. aureus* MFP03 and *S. epidermidis* MFP04, as shown in Figure 5D. Specifically, *S. aureus* CIP 107093 biofilms had an average biovolume of 9.5 μm^3^/μm^2^, compared to 3 μm^3^/μm^2^ for the other strains. Interestingly, Figures 5E,F revealed that protein and eDNA levels were significantly lower in *S. aureus* CIP 107093 biofilms than in those of *S. aureus* MFP03 and *S. epidermidis* MFP04, while polysaccharide content showed no significant differences (Figure 5G).

To assess proteolytic activity, we used skim-milk agar plates, where clear halos surrounding bacterial colonies indicated casein hydrolysis and thus enzymatic activity. Among the three tested strains, *S. aureus* CIP 107093 exhibited significantly higher proteolytic activity, as evidenced by the formation of a substantially larger halo (Figures 5H–K). In contrast, the other two strains showed little to no detectable casein hydrolysis. This marked difference implies that *S. aureus* CIP 107093 either secretes a greater quantity of extracellular proteases or produces enzymes with higher activity, under the tested conditions.

### DnaK modulates the *Staphylococcus aureus* CIP 107093 biofilm proteome, balancing stress response and metabolic regulation

**Figure 6:**
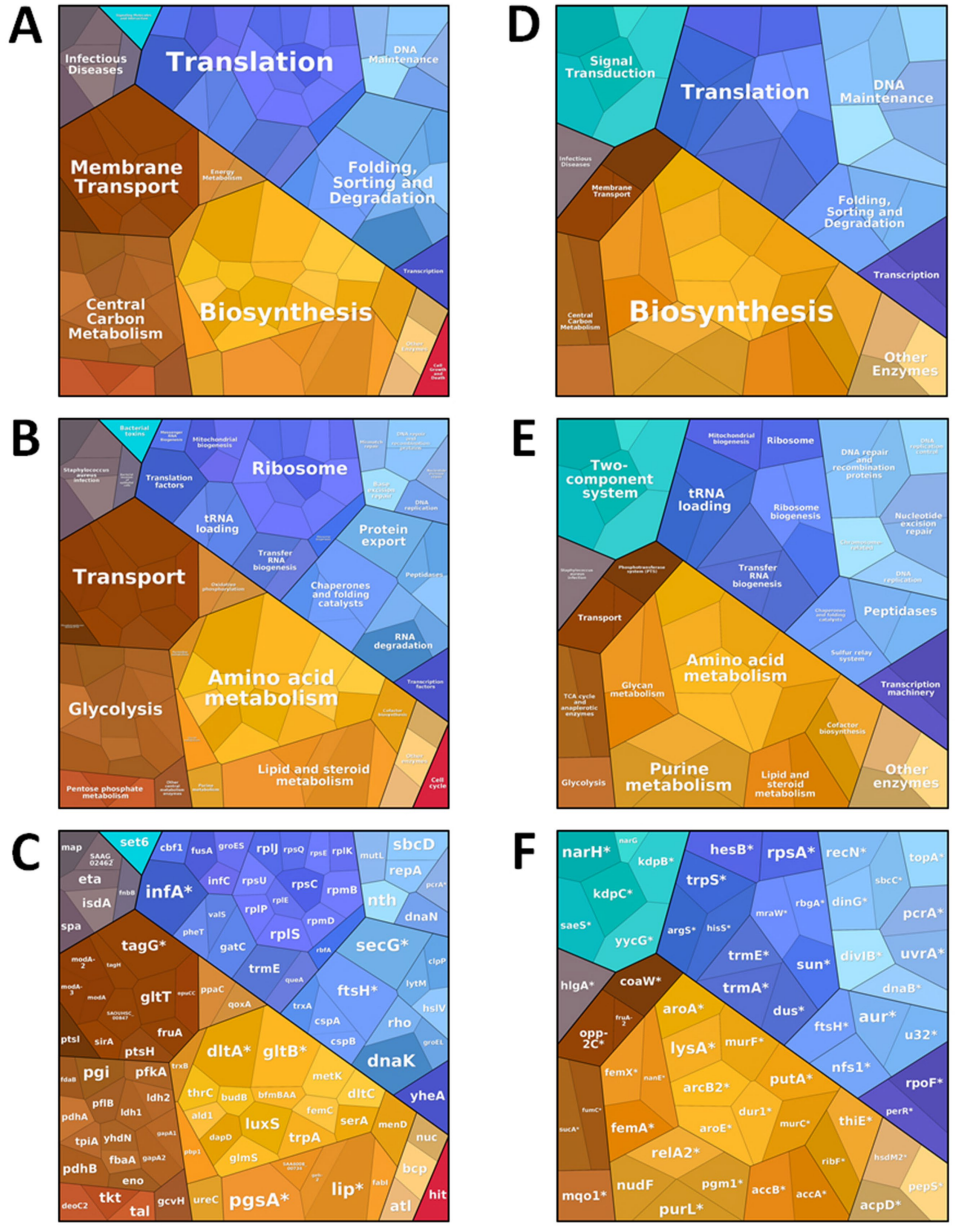
Proteomic remodeling of *Staphylococcus aureus* CIP 107093 biofilm by *Staphylococcus epidermidis* DnaK. The *Staphylococcus aureus* CIP 107093 biofilms were grown in the presence or absence of 1 μM rSep-DnaK in TSB at 37 °C for 24 h. **(A–F)** Total biofilm proteins from each condition were extracted and analyzed by LC–MS/MS. Voronoi treemaps were generated to visualize proteins that were present and more abundant **(A–C)**, and proteins that were less abundant or absent **(D–F)** in *Staphylococcus aureus* CIP 107093 biofilms treated with DnaK compared to untreated biofilms. Proteins are organized hierarchically according to the KEGG database: top-level categories **(A,D)**, second-level categories **(B,E)**, and third-level categories **(C,F)**. Colors represent functional categories, and the area of each cell corresponds to the fold change between conditions. For proteins present or absent (marked with an *), the fold change was arbitrarily set to 10.

To elucidate the molecular mechanisms underlying DnaK-mediated inhibition of biofilm formation in *S. aureus* CIP 107093, we performed an unbiased mass spectrometry-based proteomic analysis comparing protein abundance between untreated biofilms and biofilms exposed to recombinant *S. epidermidis* DnaK (Supplementary Figure S6). *S. aureus* proteins were first classified into functional groups based on KEGG annotation. Our quantitative proteomic data were then analyzed for Gene Ontology (GO) term enrichment (Supplementary Figure S7) and represented using proteomaps with Voronoi treemaps (Liebermeister et al., 2014) (Figures 6A–F).

Interestingly, the presence of DnaK significantly altered the biofilm proteome, leading to the stabilization or overexpression of several key protein families (Figures 6A–C; Table 1; Supplementary file 1). Proteins related to folding, sorting, and degradation (Table 1) were particularly enriched, including the ClpP protease, a key player in bacterial protein quality control. Notably, ClpP works alongside DnaK to degrade misfolded proteins, a process crucial for cellular proteostasis under stress conditions (Chastanet et al., 2003). Likewise, the serine-type endopeptidase ETA protease was also more abundant, indicating a broader activation of the cellular degradation machinery. In addition to proteostasis, proteins involved in central carbon metabolism, membrane transport, and infectious diseases were also more abundant in DnaK-treated biofilms. Hence, DnaK contributes not only to protein quality control but also to the metabolic adaptation of the biofilm, with potential implications for biofilm physiology and structural integrity.

**Table 1:** Relative abundance of a subset of folding, sorting, and degradation proteins differentially expressed by *Staphylococcus aureus* CIP 107093 biofilms treated with DnaK and untreated biofilms.

Uniprot	Protein	Description	Log2R*	p†
A0A0D1HKA6	DnaK	Chaperone protein	7.78	1.2E-04
A0A6B5T831	GroL	Chaperonin	2.73	3.9E-02
Q6GEI9	RpsC	Small ribosomal subunit protein	3.48	4.0E-02
A0A2S6D0C0	RplE	Large ribosomal subunit protein	2.36	4.9E-02
A0A8D9VYE6	Tig	Trigger factor	3.17	4.0E-02
A0AA86CRM4	ClpP	ATP-dependent Clp protease proteolytic subunit	2.78	4.4E-02
A0A6B5LU13	ETA	Serine protease	2.65	3.7E-02

* Log2R = Log2[DnaK-treated biofilm]/[Untreated biofilm].

†Adjusted *p*-value.

In contrast, several proteins typically enriched in mature biofilms were either absent or significantly less abundant in DnaK-treated biofilms (Figures 6D–F; Table 2; Supplementary file 2). Notably, key regulatory proteins involved in signal transduction, such as SaeS, WalK, and PerR (Table 2), were absent from the treated samples. SaeS and WalK are part of two-component regulatory systems central to *S. aureus* biofilm development and virulence regulation. SaeRS positively regulates the expression of adhesins and virulence factors, while WalKR contributes to cell wall homeostasis and biofilm maintenance (DelMain et al., 2020; Dubrac et al., 2007). Interestingly, in *S. aureus*, SaeS was reported to contribute to biofilm formation by repressing the production of extracellular proteases, which would otherwise degrade key surface-associated proteins essential for biofilm development (Mrak et al., 2012). Moreover, PerR primarily regulates oxidative stress resistance and iron homeostasis (Horsburgh et al., 2001), but no direct role of PerR in biofilm formation has yet been reported. In addition, the general stress response sigma factor SigB was not detected in DnaK-treated biofilms, which is consistent with its previously described essential role in biofilm formation in *Staphylococcus aureus* (Singh et al., 2012; Rachid et al., 2000).

**Table 2:** Relative abundance of a subset of proteins involved in signal transduction and differentially expressed by *Staphylococcus aureus* CIP 107093 biofilms treated with DnaK and untreated biofilms.

Uniprot	Protein	Description	Log2R*	p†
H9BRP9	SaeS	Histidine kinase	–	–
V5YQR2	WalK	Sensor protein kinase	–	–
Q6GGD6	–	Putative pyruvate, phosphate dikinase regulatory protein	–	–
A0A8D9SIB1	–	RNA polymerase sigma-B factor	–	–
A0A6B5LMQ1	–	PTS mannose transporter subunit IIABC	2.97	4.2E-02
Q9RQL3	PerR	Peroxide-responsive repressor	–	–

* Log2R = Log2[DnaK-treated biofilm]/[Untreated biofilm]. A dash (−) indicates absence of the protein in treated samples.

†Adjusted *p*-value.

## Discussion

*Staphylococcus aureus* and *Staphylococcus epidermidis* are Gram-positive bacteria commonly found in the human skin microbiota. In a previous study, we demonstrated that DnaK is overexpressed in the secretome of virulent *CGRP*-activated *S. epidermidis* MFP04 (N'Diaye et al., 2016). Furthermore, Singh et al. reported that the deletion of *dnaK* in *S. aureus* resulted in several physiological alterations, including increased sensitivity to heat and oxidative stress, as well as a reduction in the expression of surface-binding proteins. This ultimately led to decreased adhesion to lung epithelial cells, impaired biofilm formation, and a reduced rate of autolysis (Singh et al., 2012). Several intracellular proteins have been characterized as bacterial moonlighting proteins, acting as adhesion factors (Jeffery, 2018). Similarly, DnaK has been shown to interact with extracellular matrix proteins such as fibronectin, laminin, and collagen, and plays roles in inhibiting plasma alkaline phosphatase in *Francisella tularensis* and modulating macrophage polarization in *Mycobacterium tuberculosis* (Li et al., 2022; Arulanandam et al., 2012; Prados-Rosales et al., 2011; Lopes et al., 2014). We therefore hypothesized that extracellular DnaK from *S. epidermidis* could regulate biofilm formation in *S. aureus*. Although the structure and function of *E. coli* DnaK have been extensively characterized, with its intracellular activity well documented (Rohland et al., 2022; Kityk et al., 2012; Zhu et al., 1996), the functional role of staphylococcal DnaK in the extracellular environment remains unexplored.

Here, we expressed and purified recombinant *S. epidermidis* DnaK (rSep-DnaK) in *E. coli*, along with two mutated variants, S397P and T173A, designed to investigate the roles of the substrate-binding domain (SBD) and nucleotide-binding domain (NBD) in biofilm formation, respectively. Notably, the addition of rSep-DnaK reduced biofilm formation in the clinical strain *S. aureus* CIP 107093, while enhancing biofilm formation in *S. aureus* MFP03 and *S. epidermidis* MFP04. This strain-dependent functional variability indicates that distinct molecular contexts modulate DnaK activity. In MFP03 and MFP04, DnaK may enhance biofilm formation by stabilizing matrix-associated proteins or modulating regulatory networks that favor biofilm development, whereas in the CIP 107093 strain, DnaK likely operates through antagonistic pathways that counteract biofilm formation. In *S. aureus* CIP 107093, both the S397P and T173A mutations impaired DnaK’s ability to reduce biofilm formation, whereas neither mutation affected biofilm formation in *S. aureus* MFP03 and *S. epidermidis* MFP04. To our knowledge, this represents the first functional evidence of the involvement of DnaK’s substrate-binding domain (SBD) and nucleotide-binding domain (NBD) in extracellular processes, specifically biofilm formation. This finding indicates that DnaK exerts extracellular chaperone activity, potentially influencing biofilm dynamics through interactions with extracellular components. As biofilm formation exhibited strain-dependent variations in response to DnaK, we sought to characterize the structure and composition of these biofilms to understand the mechanisms underlying these differences. To further investigate the biofilm matrices, we employed fluorescent dyes to stain key biofilm components, followed by confocal laser scanning microscopy (CLSM). Our results revealed that the biofilm formed by *S. aureus* CIP 107093 contained proportionally less protein and extracellular DNA (eDNA) compared to the biofilms of the non-pathogenic strains MFP03 and MFP04, despite having a higher overall biovolume. However, the polysaccharide content was similar across all three biofilms. Since *S. aureus* CIP 107093 formed biofilms with relatively low protein content compared to other strains and is known for its protease activity associated with ETA toxin expression, we next assessed the proteolytic activity of these three strains. Experiments on skim-milk agar confirmed that *S. aureus* CIP 107093 exhibited significantly higher proteolytic activity, reflecting enhanced activity of extracellular proteases. Building on these findings, we aimed to molecularly characterize the proteomic changes in the biofilms of *S. aureus* CIP 107093 following treatment with the rSep-DnaK protein. Proteomic analyses revealed that DnaK modulates biofilm protein composition. In particular, DnaK-treated biofilms exhibited a marked upregulation of proteins involved in protein folding and degradation, underscoring its significant influence on the overall biofilm proteome. Key protein families, including the serine-type endopeptidase ETA protease and the ATP-dependent ClpP protease, were present in higher abundances. ClpP has been previously reported as a critical mediator of biofilm dispersal in *S. aureus*, primarily through the activation of the Agr quorum-sensing system, which leads to increased production of extracellular proteases that degrade the biofilm matrix (Liu et al., 2017). This mechanism is consistent with the decreased biofilm biomass observed in DnaK-treated samples where ClpP is overexpressed. In *S. aureus*, the transcriptional repressor CtsR regulates *clpP* and *dnaK* expression under optimal conditions (Chastanet et al., 2003). While CtsR activity was not assessed in this study, its potential role in mediating the effects of extracellular DnaK on biofilm formation remains to be determined. The antagonistic interplay between *S. epidermidis* and *S. aureus* within the skin microbiota involves proteolytic activities. While *S. epidermidis* produces serine proteases that target *S. aureus* biofilm matrix components, contributing to its inhibition (Iwase et al., 2010), our data reveal that *S. aureus* itself upregulates the serine-type endopeptidase ETA in response to DnaK treatment. Given that the orthologs DnaK and Hsp90 directly interact in *E. coli*, cooperating in client protein remodeling (Genest et al., 2011), the potential involvement of additional chaperone systems in biofilm regulation among staphylococci warrants further investigation. In addition to proteostasis, DnaK treatment increased the abundance of proteins involved in central carbon metabolism, membrane transport, and infectious diseases. This indicates that DnaK not only stabilizes protein quality control but also modulates core metabolic processes, potentially altering the biofilm’s metabolic state and structural integrity, thereby enhancing its adaptability to environmental changes. Conversely, we observed a significant reduction in the abundance of several proteins typically associated with biofilm development, including key regulatory proteins involved in signal transduction, such as SaeS and WalK (DelMain et al., 2020; Dubrac et al., 2007). The decreased levels of these proteins indicate that DnaK interferes with important regulatory networks, potentially disrupting the finely tuned processes that govern biofilm formation and maintenance. The mechanism by which DnaK influences these regulators remains unclear but could involve direct protein–protein interactions or indirect effects through proteostasis modulation. Finally, SigB, an alternative sigma factor central to stress response and biofilm regulation, was undetectable in biofilms treated with DnaK. The absence of detectable SigB, which correlates with the observed reduction in biofilm thickness, is consistent with previous studies demonstrating the essential role of SigB in promoting biofilm formation in *S. aureus* (Singh et al., 2012; Rachid et al., 2000). While our data reveal a clear strain-dependent modulation of biofilm formation by *S. epidermidis* DnaK, extending this analysis to a larger set of clinical *S. aureus* isolates will be essential to assess whether this effect reflects a general response to pathogenic strains.

The ability of DnaK to alter biofilm architecture and metabolic pathways suggests a broader role in microbiota homeostasis, potentially influencing interactions between commensal and opportunistic species. Beyond its role in biofilm modulation, DnaK may also modulate microbial interactions within the skin microbiota. These findings raise the possibility that extracellular DnaK functions as an additional layer of signaling, influencing bacterial community dynamics.

## Data Availability

The datasets presented in this study can be found in online repositories. The names of the repository/repositories and accession number(s) can be found in the article/Supplementary material.
