# Supplementary material for: Staphylococcus epidermidis DnaK alters biofilm formation and proteome in Staphylococcus aureus CIP 107093
Source: Front Microbiol. 2026 Feb 18;16:1705130. doi: 10.3389/fmicb.2025.1705130 (PMC12957280; doi:10.3389/fmicb.2025.1705130)
Supplement: Supplementary file 1 [file Data_Sheet_1.docx]

**Supplementary materials**

**Supplementary Figure 1: Purification of recombinant DnaK-WT and variants T173A and S397P.**

Representative 12% SDS–PAGE analysis of recombinant DnaK wild-type (WT) and point mutants T173A and S397P. Lane MW: molecular weight marker. (A) Proteins following Ni²⁺-affinity chromatography and elution with 250 mM imidazole. (B) Purified proteins after size-exclusion chromatography.

**Supplementary Figure 2: Comparative growth kinetics of *S. aureus* CIP 107093 (A), *S. aureus* MFP03 (B), and *S. epidermidis* MFP04 (C) strains.**

Bacteria were cultured for 24 hours in TSB supplemented with 0.5% glucose in the presence of 1 µM rSep-DnaK-WT (triangles) or BSA (squares). Untreated bacteria are represented by circles. Data are shown as mean values (± SEM), representing three independent experiments.

**Supplementary Figure 3: Primary sequence alignment and domain organization of DnaK.**

Multiple sequence alignment of the DnaK proteins from *E. coli* K12, *S. aureus* MFP03, *S. aureus* CIP 107093, and *S. epidermidis* MFP04. The alignment includes the annotated secondary structure elements of *E. coli* DnaK (α-helices, β-strands shown as arrows, and turns labeled as “TT”) displayed above the sequences*.* Identical residues across all three sequences are shaded in blue, and highly conserved residues (>70% similarity) are shown in blue text. Blue and red highlights indicate functionally validated point mutations in *E. coli* DnaK, as previously reported **(52-54)**, which informed the design of corresponding substitutions at conserved sites in *S. epidermidis* DnaK in the present study. The alignment was generated using the ESPript 3.0 server.

**Supplementary Figure 4: Structural conservation between DnaK from *Escherichia coli* K12 and *Staphylococcus epidermidis* MFP04.**

(A) Structural comparison of full-length DnaK structures from *E. coli* K12 (PDB ID: 4B9Q, shown in blue) and *S. epidermidis* (AlphaFold model AF3, shown in orange).

(B) Close-up view of the Nucleotide-Binding Domain (NBD), highlighting structural conservation between the two homologs.

(C) Structural comparison of the alpha subdomain of the substrate-binding domain (SBD-α), showing overall conservation of the helical bundle despite variations at the C-terminal region.

(D) Structural alignment of the beta subdomain of the substrate-binding domain (SBD-β) showing strong conservation in β-sheet topology and substrate interaction surfaces.

NBD: nucleotide-binding domain; SBD: substrate-binding domain; SBD-α: alpha subdomain; SBD-β: beta subdomain

**Supplementary Figure 5: Biofilm formation of *S. aureus* CIP 107093 treated with rSep-DnaK-WT and peptides NRLLLTG and CGRP.**

Biofilms were cultured in TSB medium (0.5% glucose) for 24 hours at 37°C, and treated with either rSep-DnaK protein or BSA (set as 100% control), in the presence or absence of the peptides NRLLLTG or CGRP. Protein and peptide concentrations were maintained at 1 µM. Data are presented as mean values (± SEM), representing three independent experiments. Statistical significance was determined by one-way ANOVA with Tukey’s post hoc test, with p-value: ****p < 0.0001.

**Supplementary Figure 6: Differential proteomic analysis of DnaK-treated and untreated *S. aureus* biofilms.**

Volcano plots displaying proteins significantly enriched or depleted upon DnaK treatment (BF_DnaK) compared to untreated biofilms (BF). Significance was determined using a two-sided t-test with a false discovery rate (FDR) threshold of 5% and a fold change cutoff of 2. Side histograms indicate the iBAQ values (intensity-based absolute quantification) for proteins exclusively detected in one condition, providing an estimate of their relative abundance.

**Supplementary Figure 7: Functional enrichment analysis of proteins modulated by DnaK in *S. aureus* CIP 107093.**

GO term distributions of proteins that were less abundant or absent in the presence of DnaK (left) or more abundant or uniquely detected with DnaK (right) were compared to the complete proteome using a hypergeometric test. GO terms with p-value inferior to 0.05 are shown; circle size indicates the number of proteins associated with each GO term, and color intensity reflects the statistical significance.

Supplementary Figure 1


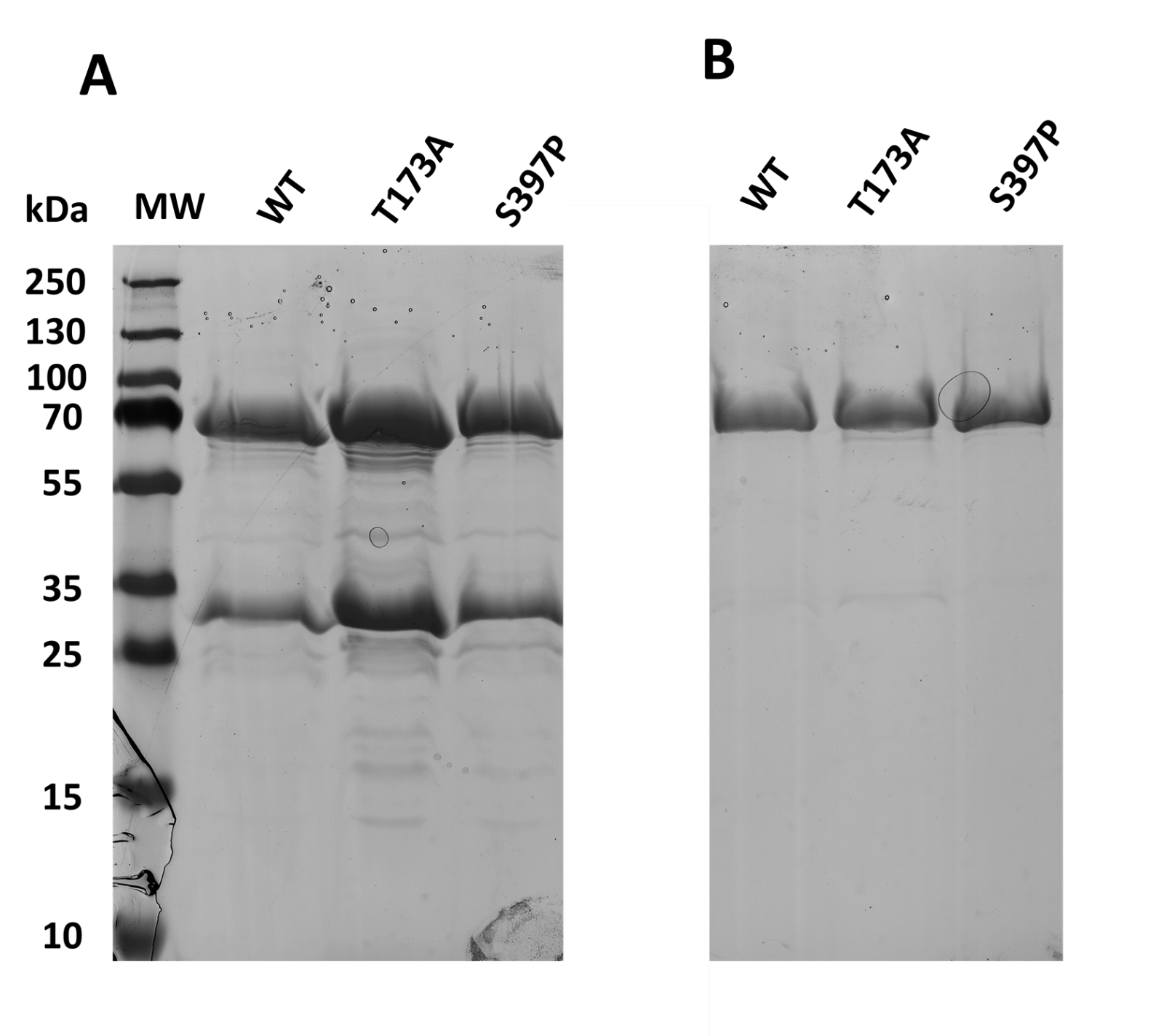


Supplementary Figure 2


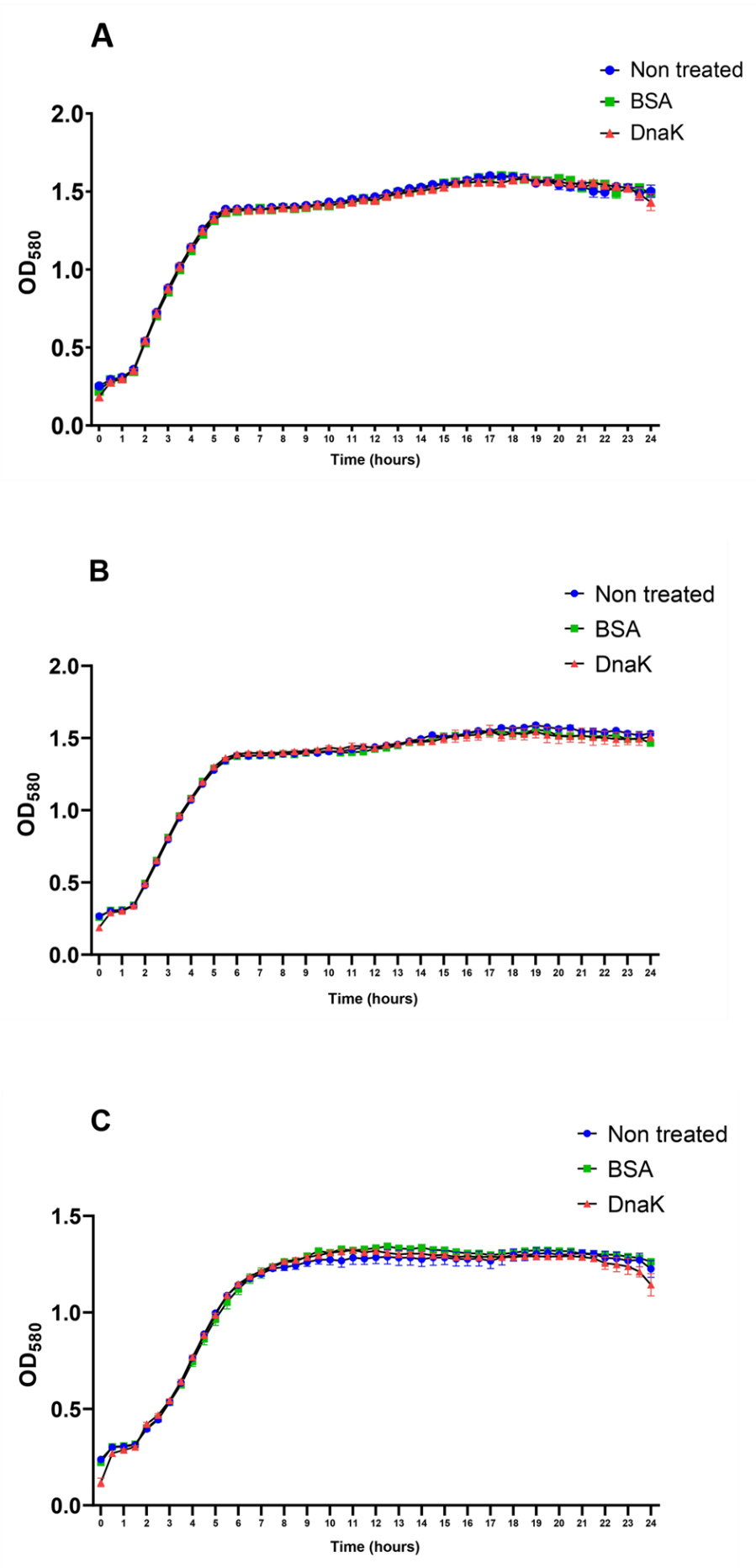


Supplementary Figure 3


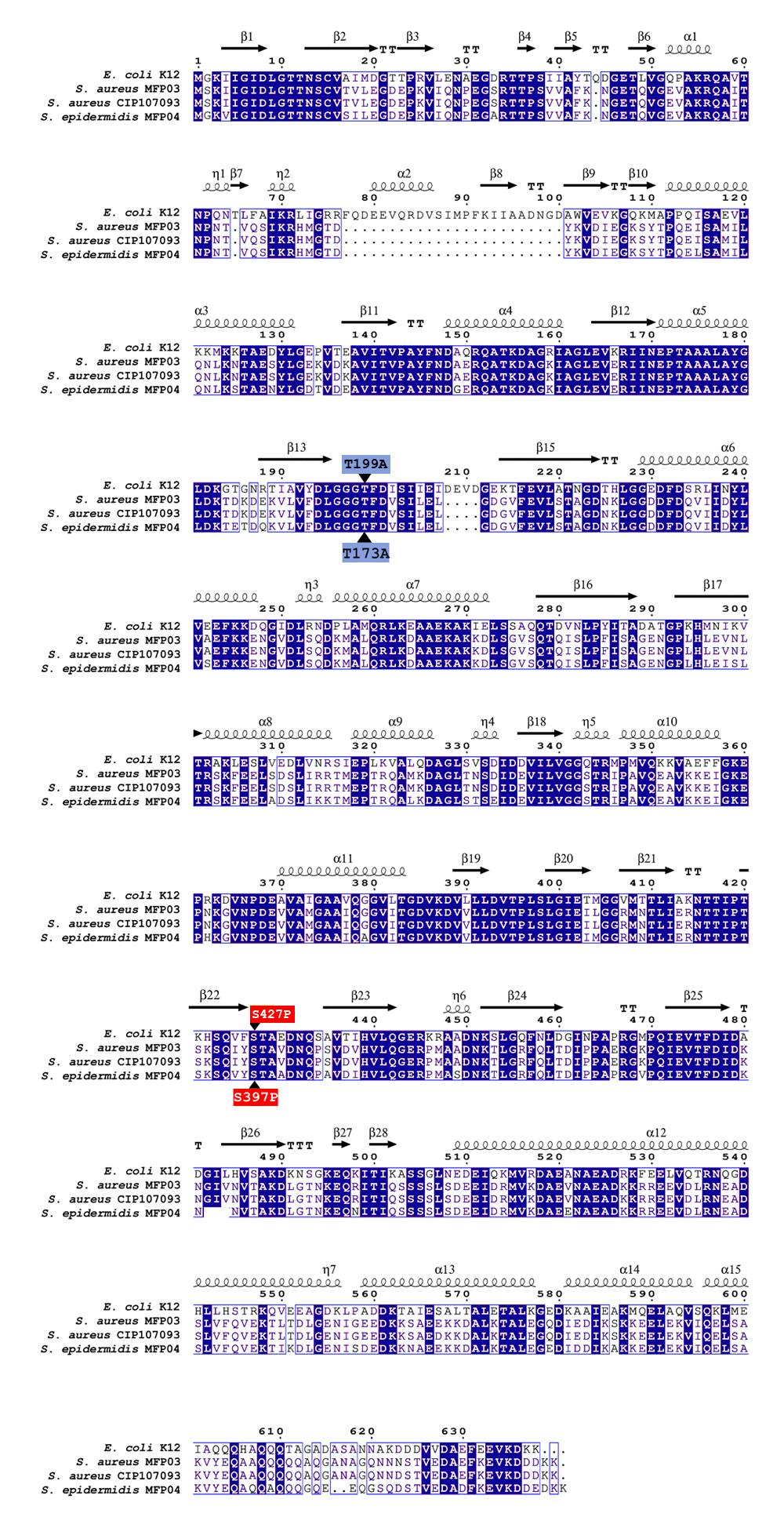


Supplementary Figure 4


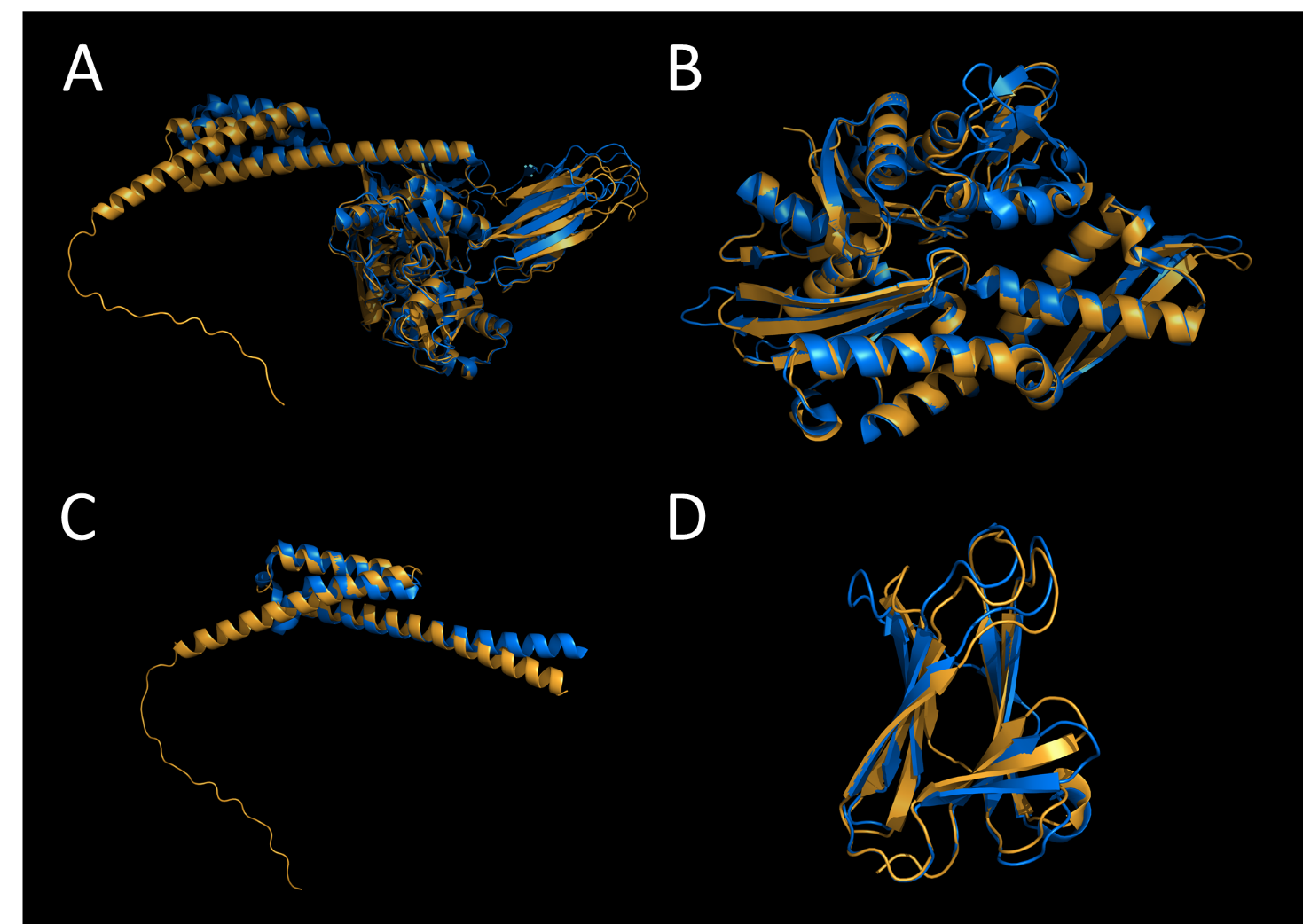


Supplementary Figure 5


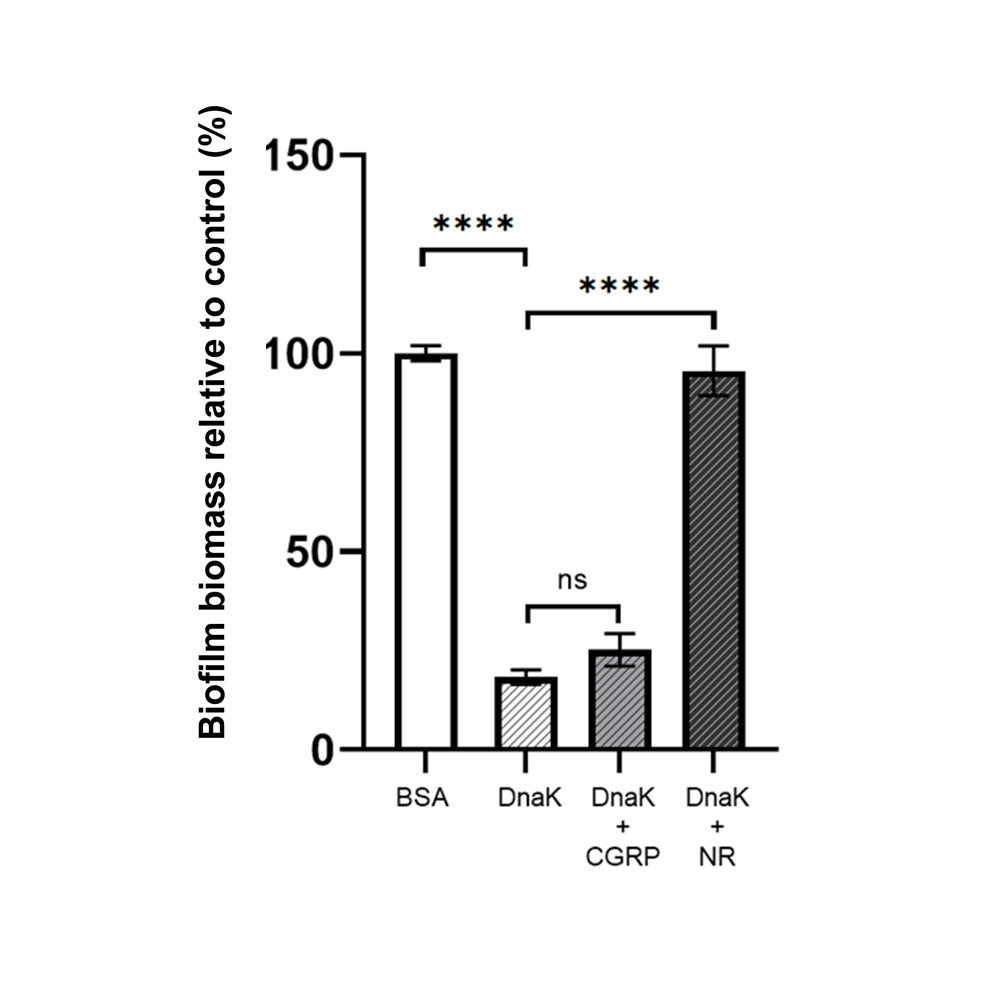


Supplementary Figure 6


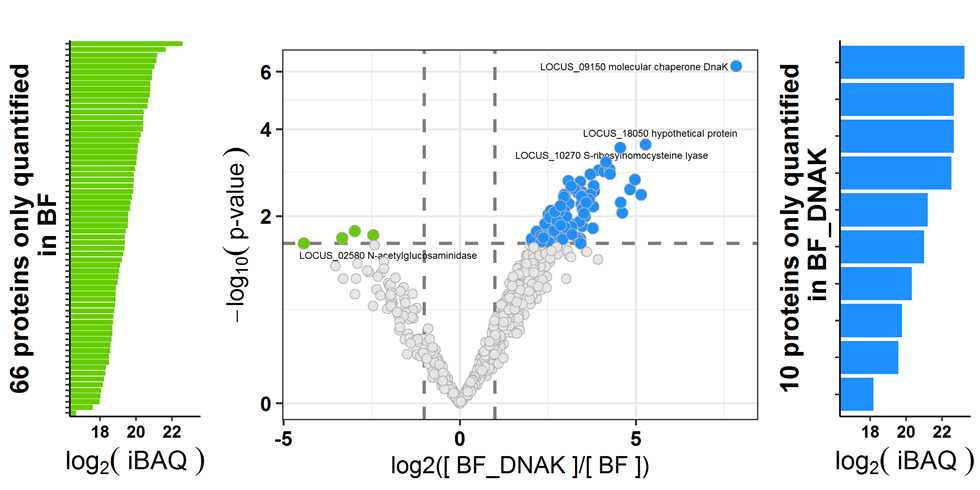


Supplementary Figure 7


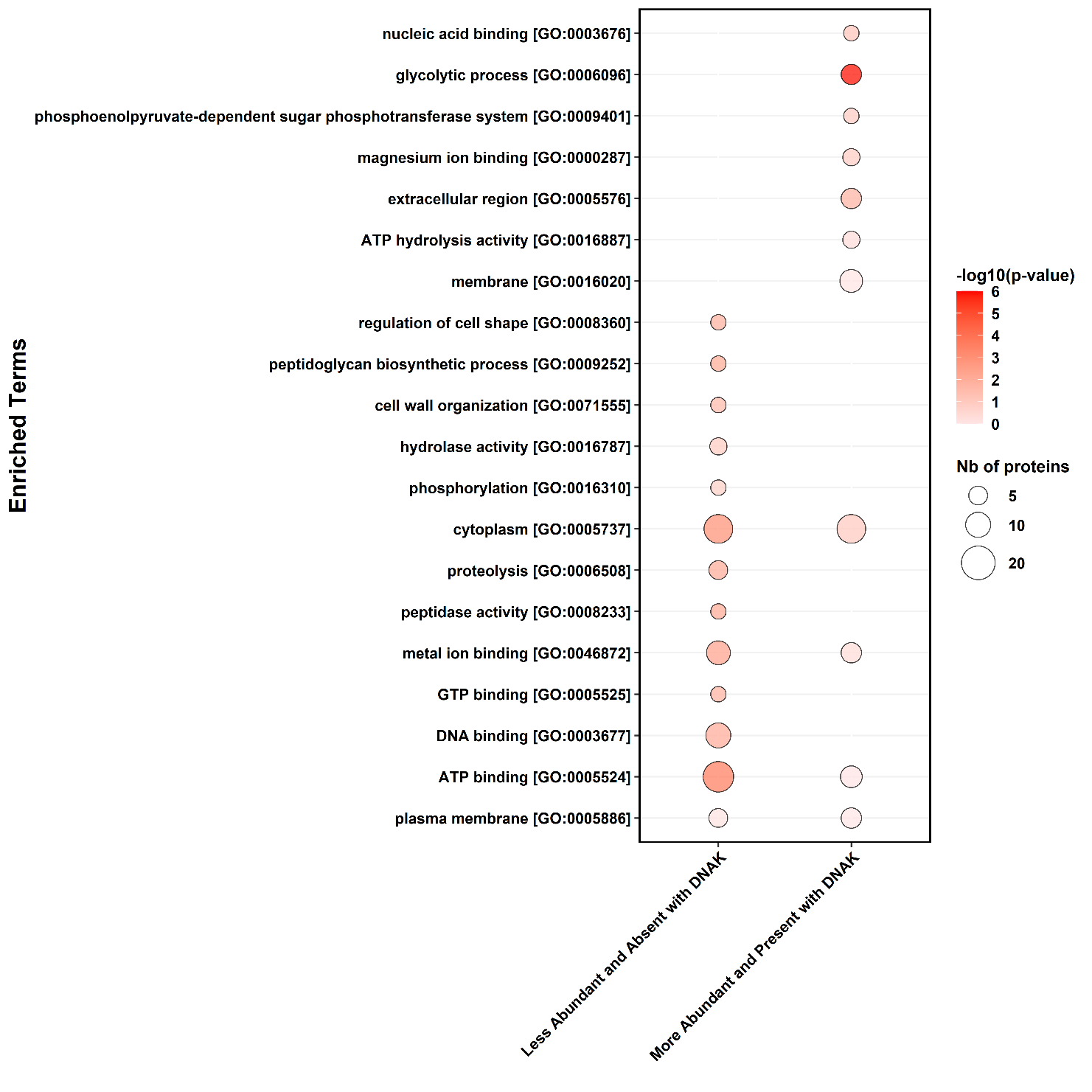


[Supplementary Table 1](https://www.ncbi.nlm.nih.gov/pmc/articles/PMC7752777/#SM1)

Comparative analysis of *E. coli* K12 DnaK (PDB ID: 4B9Q) with the AlphaFold model AF3 of *Staphylococcus epidermidis* MFP04 DnaK. The structural alignments and the RMSD calculations were performed using PyMOL


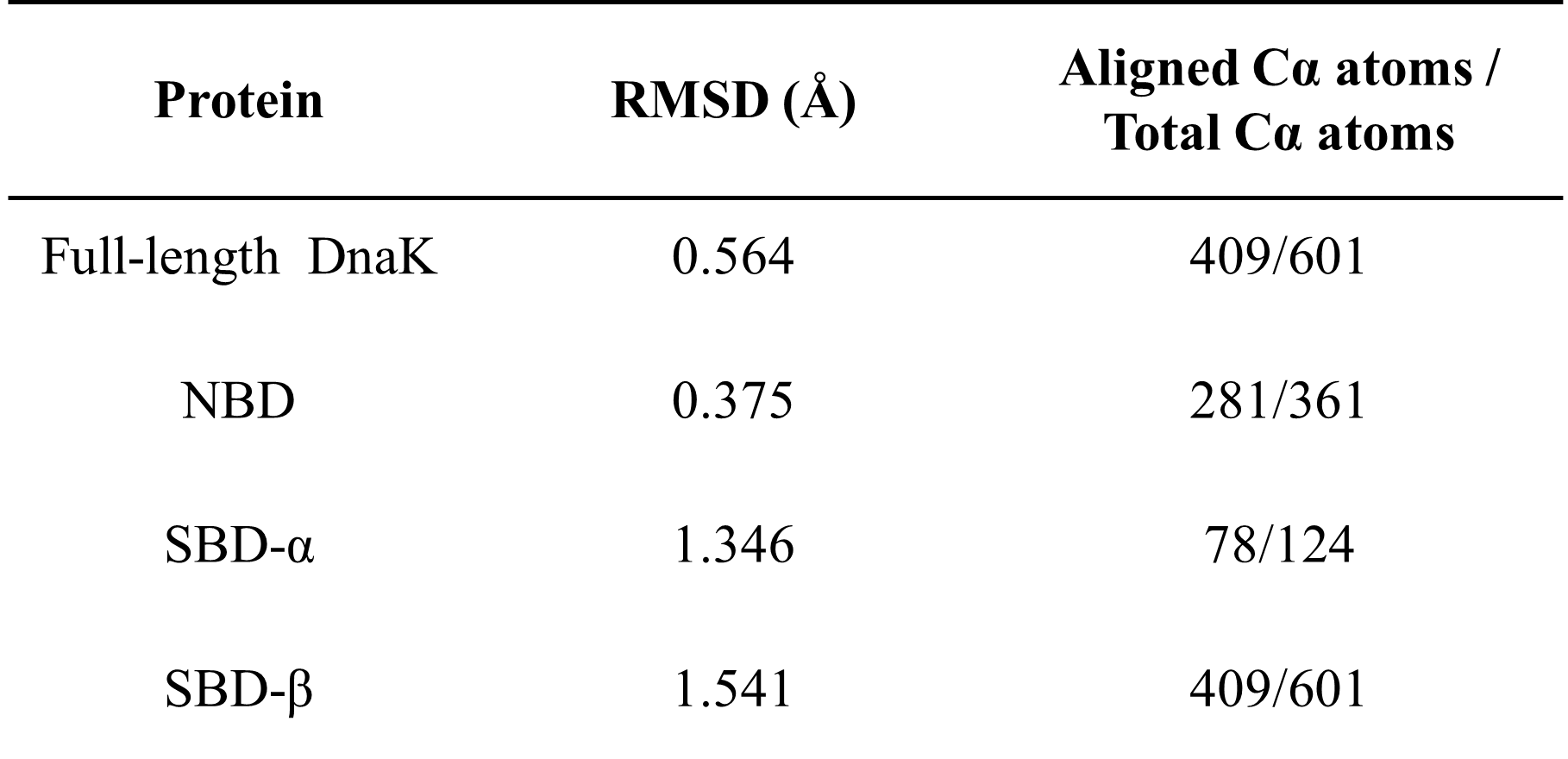


[Supplementary Table 2](https://www.ncbi.nlm.nih.gov/pmc/articles/PMC7752777/#SM1)

Bacterial strains and plasmids used in this study

| **Strain or plasmid** | **Characteristics** | **Reference** |
| --- | --- | --- |
| *Staphylococcal strains* | | |
| CIP 107093 | *Staphylococcus aureus* isolated from skin | (36) |
| MFP03 | *Staphylococcus aureus* isolated from skin | (35) |
| MFP04 | *Staphylococcus epidermidis* isolated from skin | (35) |
| *Escherichia coli strains* | | |
| DH5α | *supE44 ΔlacU169 (Φ80lacZΔM15) hsdR17 recA1 endA1 gyrA96 thi-1 relA1* | Lab collection |
| BL21(λDE3) | F^-^*ompT hsdSB(rB-mB-)gal dcm* | Lab collection |
| BL21(λDE3)/pET22-*dnaK-WT* | BL21(λDE3) strain carrying plasmid pET22-*dnaK-WT* | This study |
| BL21(λDE3)/pET22-*dnaK-T173A* | BL21(λDE3) strain carrying plasmid pET22-*dnaK-T173A* | This study |
| BL21(λDE3)/pET22-*dnaK-S397P* | BL21(λDE3) strain carrying plasmid pET22-*dnaK-S397P* | This study |
| *Plasmids* | | |
| pUC19-*dnaK* | *dnaK* ORF amplified and cloned into pET22b+ |  |
| pET22-*dnaK-WT* | *dnaK-WT* ORF amplified and cloned into pET22b+ | This study |
| pET22-*dnaK-T173A* | *dnaK-T173A* ORF amplified and cloned into pET22b+ | This study |
| pET22-*dnaK-S397P* | *dnaK-S397P* ORF amplified and cloned into pET22b+ | This study |

[Supplementary Table 3](https://www.ncbi.nlm.nih.gov/pmc/articles/PMC7752777/#SM1)

Primers used in this study.

| **Primer** | **Sequence (5’–3’)** |
| --- | --- |
| **Fwd-long-MFP04** | CATCAAGCTGTCGTTCAAGATGACAATCC |
| **Rev-long-MFP04** | AGAAGCGCTTTTGCTTACGCCTAA |
| **M13F** | GTAAAACGACGGCCAG |
| **M13R** | CAGGAAACAGCTATGAC |
| **T210C-fwd** | AAACGTCAcATGGGTACAGATTATAAAGTAGAT |
| **T210C-rev** | ACCCATgTGACGTTTAATAGATTGTACAGTGT |
| **NdeI-*dnaK*** | ATTATcatATGggtaaagtaattggaattgatttaggtacaactaactc |
| **XhoI-*dnaK*** | actaCTCGAGttttttatcttcgtcatctttaacttctttaaagtctgc |

Supplementary File 1

Proteins more abundant in *S. aureus* CIP 107093 biofilms treated with DnaK, compared to untreated biofilms.

MoreAbinBF-DnaK_than_BF.xlsx

Supplementary File 2

Proteins less abundant in *S. aureus* CIP 107093 biofilms treated with DnaK, compared to untreated biofilms.

MoreAbinBF_than_BF-DnaK.xlsx

Supplementary File 3

Proteomaps input data for DnaK-treated and untreated *S. aureus* biofilms

Proteomaps_data.xlsx
